# Randomized Trial Evaluating the Impact of Ribavirin Mono-Therapy and Double Dosing on Viral Kinetics, Ribavirin Pharmacokinetics and Anemia in Hepatitis C Virus Genotype 1 Infection

**DOI:** 10.1371/journal.pone.0155142

**Published:** 2016-05-11

**Authors:** Jesper Waldenström, Johan Westin, Kristina Nyström, Peer Christensen, Olav Dalgard, Martti Färkkilä, Karin Lindahl, Staffan Nilsson, Gunnar Norkrans, Henrik Krarup, Hans Norrgren, Mads Rauning Buhl, Stephan Stenmark, Martin Lagging

**Affiliations:** 1 Department of Infectious Medicine, Institute of Biomedicine at Sahlgrenska Academy, University of Gothenburg, Gothenburg, Sweden; 2 Department of Infectious Diseases, University of Southern Denmark, Odense, Denmark; 3 Department of Infectious Diseases, Akershus University Hospital, Oslo, Norway; 4 Department of Gastroenterology, Helsinki University, Helsinki, Finland; 5 Department of Infectious Diseases, Karolinska University Hospital Huddinge, Karolinska Institute, Stockholm, Sweden; 6 Department of Mathematical Sciences, Chalmers University of Technology, Gothenburg, Sweden; 7 Section of Molecular Diagnostics, Clinical Biochemistry, Aalborg University Hospital, Aalborg, Denmark; 8 Department of Infectious Diseases, Skåne University Hospital, Lund, Sweden; 9 Department of Infectious Diseases, Aarhus University, Aarhus, Denmark; 10 Department of Communicable Disease Control Västerbotten, Umeå, Sweden; University of New South Wales, AUSTRALIA

## Abstract

In this pilot study (RibaC), 58 hepatitis C virus (HCV) genotype 1 infected treatment-naïve patients were randomized to (i) 2 weeks ribavirin double dosing concomitant with pegylated interferon-α (pegIFN-α), (ii) 4 weeks ribavirin mono-therapy prior to adding pegIFN-α, or (iii) standard-of-care (SOC) ribavirin dosing concurrent with pegIFN-α. Four weeks of ribavirin mono-therapy resulted in a mean 0.46 log_10_ IU/mL HCV RNA reduction differentially regulated across *IL28B* genotypes (0.89 vs. 0.21 log_10_ IU/mL for CC and CT/TT respectively; P = 0.006), increased likelihood of undetectable HCV RNA week 4 after initiating pegIFN-α and thus shortened treatment duration (P<0.05), and decreased median IP-10 concentration from 550 to 345 pg/mL (P<0.001). Both experimental strategies impacted on ribavirin concentrations, and high levels were achieved after one week of double dosing. However, by day 14, double dosing entailed a greater hemoglobin decline as compared to SOC (2.2 vs. 1.4 g/dL; P = 0.03). Conclusion: Ribavirin down-regulates IP-10, and may have an anti-viral effect differently regulated across *IL28B* genotypes.

**Trial Registration:**

ClinicalTrials.gov NCT01226771

## Introduction

Therapy for hepatitis C virus (HCV) infection recently has undergone major improvements regarding therapeutic efficacy and reduction of side effects following the introduction of interferon-free regimens comprising combinations of direct acting antivirals (DAA) [1–12], often including the use of ribavirin. Presently ribavirin is recommended for several DAA-based treatments for HCV genotype 1–3 infection [13], and likely will remain a vital component of therapy.

Ribavirin is a purine nucleoside analogue with broad-spectrum *in vitro* antiviral activity against many RNA and DNA viruses [14]. In addition to its utility in HCV therapy, ribavirin also is administered for severe respiratory syncytia virus (RSV) infections [15], viral hemorrhagic fevers such as Lassa and Crimean–Congo [16, 17], and hepatitis E virus infections [18]. Unaided, ribavirin is insufficient to achieve clearance of HCV infection [19, 20], but in interferon-based combination therapy, it is pivotal to increase the likelihood of achieving a sustained virological response (SVR) by means of reduced relapse risk [21, 22]. Ribavirin mono-therapy reportedly has a modest effect on HCV RNA, with mean reductions of approximately 0.5 log_10_ IU/mL, in addition to reducing systemic concentrations of liver enzymes [23].

The underlying molecular basis through which ribavirin impacts on HCV infection remains unclear, but several mechanisms of action have been proposed; direct inhibition of the viral RNA polymerase, inhibition of inosine monophosphate dehydrogenase (IMPDH) with ensuing GTP depletion, viral mutagenesis leading to error catastrophe, and modulation of T cell responses [24]. Recently both *in vivo* [25] and *in vitro* [26, 27] studies also have suggested that ribavirin modulates the expression of interferon-stimulated genes (ISGs). The observation that genetic variants of the inosine triphosphate pyrophosphatase gene, which likely also lead to decreased GTP intracellular concentrations, are associated with a ribavirin-like reduced relapse risk following treatment for HCV genotype 2/3 [28] support the IMPDH hypothesis as the possible primary mechanism of action of ribavirin.

Ribavirin has a large distribution volume, has an elimination that is dependent on renal function, and has a long half-life requiring in excess of 4 weeks to reach steady state [29–31]. It is administered as a pro-drug, which subsequently is activated by intracellular phosphorylation to mono-, di- and triphosphates, and upon phosphorylation becomes irreversibly entrapped in erythrocytes. Higher ribavirin exposure during the early phase of treatment reportedly is important for HCV treatment response [32, 33]. However, the use of high dose treatment is hampered by enhanced side effects, most importantly a dose dependent hemolytic anemia, possibly secondary to oxidative stress caused by posterior depletion of ATP in erythrocytes [34, 35]. Recently some studies have evaluated the effect of using a lead-in phase of ribavirin mono-therapy prior to the addition of interferon, with the goal of achieving steady state concentrations early in combination-treatment without causing severe anemia; however, thus far this strategy has not been reported to impact on outcome [27, 36–38].

Several host and viral factors have been used to prognosticate HCV clearance, including genetic polymorphisms in the proximity of the interleukin 28B (*IL28B*) gene, also known as interferon-λ4 (*IFNL4*), which have been coupled to both treatment response [39] and spontaneous resolution of infection [40, 41]. Similarly, lower systemic concentrations of interferon gamma inducible protein 10 (IP-10 also known as CXCL10) are associated with successful therapeutic outcome [39, 42, 43] and spontaneous clearance [44].

The aim of the present pilot study (RibaC) was to further explore the impact on HCV viral kinetics, ribavirin pharmacokinetics and anemia of a 4-week lead-in phase of ribavirin mono-therapy prior to the addition of pegylated interferon-α (pegIFN-α), referred to as “priming”, as well as an initial 2-week double-dosage of ribavirin concomitant with pegIFN-α, referred to as “loading”, as compared to standard-of-care ribavirin and pegIFN-α therapy for chronic HCV genotype 1 infection. When the present study was designed and initiated, DAA-based regimens were not readily available, and the standard-of-care at the time was pegIFN-α and ribavirin combination therapy. Later, the standard-of-care has been revised in many regions to include the use DAAs, especially HCV polymerase inhibitors, as well as interferon-free treatments often including the use of ribavirin [13].

## Materials and Methods

### Patients

**Fig 1:**
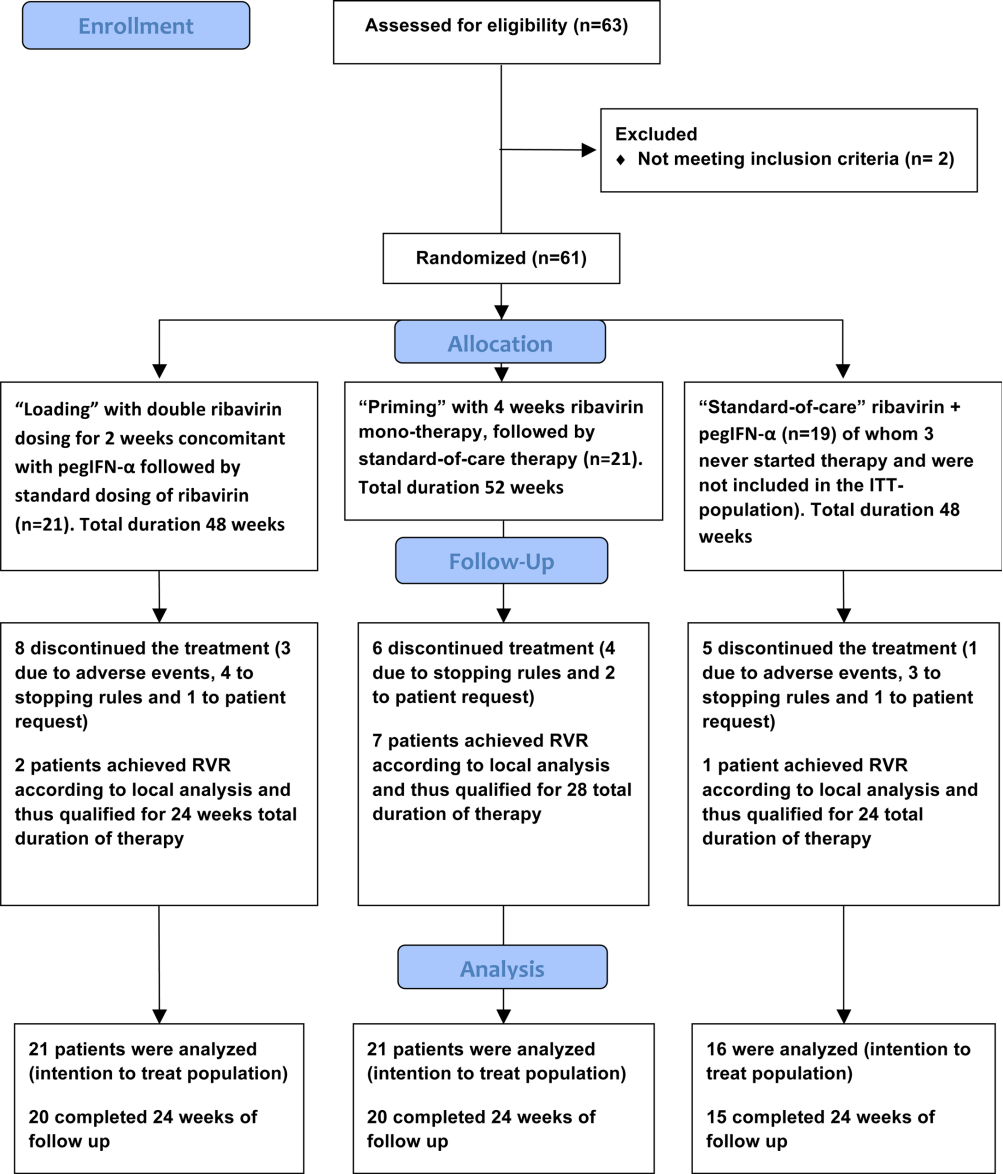
Flow diagram of the RibaC study trial showing enrollment and disposition of patients. Patients were randomized to arm A “loading” (2 weeks of ribavirin double dosing concomitant with pegIFN-α), arm B “priming” (4 weeks ribavirin mono-therapy prior to adding pegIFN-α), or arm C “standard-of-care”.

Between October 22, 2010 (after registration at ClinicalTrials.gov) and January 2012, 63 patients with chronic HCV genotype 1 infection were screened for inclusion in a randomized, open-label, parallel group, multicenter pilot study (RibaC), conducted at 11 centers in Sweden, Denmark, Finland and Norway (disposition of patients in Fig 1; study protocol in supporting information S1 File). Sixty-one enrolled patients met the inclusion criteria and 58 patients agreed to start medication and constituted the intention to treat (ITT) population. All patient where adults (≥18 years of age), had compensated liver disease (Child-Pugh clinical classification A) and were interferon treatment naïve, seronegative for hepatitis B surface antigen and HIV antibodies and met the additional inclusion criteria: Serologic evidence of chronic hepatitis C infection and serum HCV RNA ≥15 IU/mL, HCV genotype 1, confirmed within 2 years of treatment initiation. Liver biopsy or elastography were not mandatory, but encouraged. Enrollment planned to include 105 patients, 35 per treatment group. However, this number of patients could not be recruited, secondary to swift establishment of DAA-based HCV therapy.

### Treatment

Patients were randomized during the screening period between 4 to 12 weeks before treatment initiation to one of three arms; arm A “loading”, arm B “priming” and arm C “standard-of-care”. Arm A “Loading” received pegIFN α-2a 180 μg/week plus loading (≥26 mg/kg/day for 2 weeks followed by ≥13 mg/kg/day) and concentration targeted (≥2.5 mg/L (10.25 μmol/L) 28 days after initiation of pegIFN-α therapy) dosing. This target was chosen based on the finding that 2.0 mg/L (8.2 μmol/L) was sufficient in HCV genotype 2/3 [45] and that genotype 1 would require a higher goal concentration. Arm B “Priming” received standard-of-care dosing of ribavirin (≥13 mg/kg/day) without pegIFN for 4 weeks followed by 48 additional weeks of pegIFN α-2a 180 μg/week plus standard-of-care dosing of ribavirin (≥13 mg/kg/day) and concentration targeted (≥2.5 mg/L (10.25 μmol/L) 28 days after initiation of pegIFN-α therapy) dosing. Arm C “Standard-of-Care” received pegIFN α-2a 180 μg/week plus standard-of-care dosing of ribavirin (≥13 mg/kg/day). Regardless of treatment arm, all patients with undetectable HCV RNA as analyzed by their local laboratory 4 weeks after initiation of pegIFN-α received 24 weeks of combination treatment; otherwise the patient received 48 weeks. Follow up sampling was performed 24 weeks after end of treatment. Standard stopping rules were applied for treatment discontinuation if the patients had ≤2 log_10_ reduction in HCV RNA by week 12 or detectable HCV RNA at week 24, and patient meeting the stopping rules were considered as “non-responders” not having achieved SVR.

### Study endpoints

Primary endpoints in this study were the early virological responses measured by the decline in HCV RNA during the first 12 weeks of combination therapy, in particular the first and second phase decline. Secondary endpoints were to determine the evaluate differences in plasma HCV RNA, IP-10, *IL28B* genotype, ribavirin concentrations, as well as the proportion of patients achieving VRVR, RVR, cEVR, pEVR and SVR in the different treatment arms. Additional endpoints were evaluation of safety of “priming” and “loading” ribavirin-dosing regimens.

### HCV RNA quantification and genotyping

Plasma was obtained with PPT-tubes at day (-28, only in arm B), 0, 3, 7, 28, week 12, End-of-Treatment, and Follow-Up week 24. Samples were kept frozen (-70°C) and analyzed at the central lab (Gothenburg, Sweden) using Cobas 48 Taqman HCV-RNA-test (Roche Diagnostics, Branchburg, NJ) that quantifies HCV RNA with a limit of detection of ≤15 IU/mL. Genotyping of HCV was initially performed at the local centers and subsequently confirmed at the central laboratory (Gothenburg, Sweden) with a TaqMan primer-specific reverse-transcription polymerase chain reaction method.

### *IL28B* (*IFNL4*) genotyping

*rs12979860* was determined using TaqMan SNP genotyping assays (Applied Biosystems Inc., Foster City, CA) as previously described [46].

### IP-10 (CXCL10) quantification

Quantification of IP-10 was performed using Quantikine (R&D Systems, Minneapolis, MN), a solid-phase enzyme-linked immunosorbent assay, on plasma samples obtained at screening visit, days (-28, -27, -21 only in arm B), 0, 1, 3, 7, 14 and weeks 8, 18, 24. All samples were stored at −70°C until assayed at the central laboratory (Gothenburg, Sweden).

### Ribavirin concentrations

Plasma samples for ribavirin drug concentration measurement were drawn immediately before the morning dose of ribavirin, *i*.*e*. trough concentrations, at day (-27, -21, 0, only in arm B), 1, 3, 7, 14, 28, and weeks 8, 12, 18, and at end of treatment. Plasma ribavirin concentrations were measured by use of solid phase extraction and high-performance liquid chromatography (HPLC; Merck-Hitachi, Tokyo, Japan) followed by UV-detection (wavelength 215 nm).

### Statistics

Fisher’s exact test was used to evaluate differences in frequencies of SVR, and undetectable HCV RNA by week 12 and end-of-treatment. Mann-Whitney U-test was used when comparing groups and Spearman’s test for correlation analysis. Statistical analyses were performed using Prism (Version 5.0c, GraphPad Software, La Jolla, CA) or SPSS (Version 20.0.0, IBM Corp, Armonk, NY, USA) software. All reported P values are two-sided, and P values <0.05 were considered significant. The sample size calculation to demonstrate an increase in the reduction of HCV RNA from day 0 to 3 from 0.9 log_10_ IU/mL in Group C (Standard-of-Care) to 1.4 log_10_ IU/mL in Group A or B or an increase in the reduction of HCV RNA from day 7 to 28 from 0.4 log_10_ IU/mL/week in Group C (Standard-of-Care) to 0.6 log_10_ IU/mL/week in Group A or B the study required at least 35 patients per study arm, a number we were unable to recruit despite considerable efforts. The statistical power (chance) for the study to detect a superior effect in arm A or B as compared to C was 80%. Statistical sample-size calculation was based on z-test for differences between proportions and was one-sided with a significance level of 5%.

### Ethical Considerations

The treatment study conformed to the guidelines of the 1975 Declaration of Helsinki. Written informed consent was obtained from each participating patient, and ethics committees in each participating country approved the study as of May 3, 2010 (i.e. Regional Ethical Review Board, Gothenburg, Sweden (Regionala etikprövningsnämnden i Göteborg), Regional Committee for Ethics in Medical Research, Oslo, Norway (Regionaletisk komite for medisinsk og helsefaglig forskning i Oslo), The Scientific Ethical Committee for the Region of South Denmark, Vejle, Denmark (Den Videnskabsetiske Komité for Region Syddanmark), and the Ethics Committee, Department of Medicine for the Hospital District of Helsinki and Uusimaa, Finland (Etiska kommittén för invärtesmedicin)). The study also was approved by the Medical Products Agency in Sweden as of May 28, 2010.

### Clinical Trial Registration

This trial has been registered at the National Institutes of Health trial registry as of October 21, 2010 (ClinicalTrials.gov identifier: NCT01226771). Recruitment of patients began after registration. The authors confirm that all ongoing and related trials for this drug/intervention are registered.

## Results

### Baseline characteristics

The investigational arms (arm A “loading” and B “priming”) were balanced according to baseline characteristics (Table 1) compared to the “standard-of-care” (SOC) arm C (disposition of patients shown in Fig 1).

**Table 1:** Baseline Characteristics for Patients According to Treatment Arm (ITT population).

	Arm A ("Loading")	Arm B ("Priming")	Arm C ("Standard-of-Care")
	(n = 21)	(n = 21)	(n = 16)
Female gender (n (%))	9 (43%)	9 (43%)	5 (31%)
Age (years)	46.5 ± 10.5	44.2 ± 10.7	47.0 ± 8.7
Weight (kg)	82.8 ± 16.0	79.2 ± 12.7	76.6 ± 16.1
BMI (kg/m2)	27.13 ± 3.9	26.18 ± 3.5	25.35 ± 4.4
HCV Genotype (n (%))			
1a	15 (71%)	15 (71%)	12 (75%)
1b	6 (29%)	5 (24%)	3 (19%)
1 (subtyping not possible)	0 (0%)	1 (5%)	1 (6%)
Log10 HCV RNA (IU/mL)	6.51 ± 0.55	5.83 ± 0.69	6.16 ± 0.59
ALT (IU/L)	72.7 ± 34.7	87.3 ± 85.7	91.8 ± 69.2
Hemoglobin (g/dL)	147 ± 17	145 ± 14	146 ± 14
IP-10 (pg/ml)	298 (102; 4833)	339 (113; 2784)	241 (106; 2718)
*IL28B* (*IFNL4*) rs12979860 (n (%))			
CC	7 (33%)	8 (38%)	7 (44%)
CT or TT	14 (67%)	13 (62%)	9 (56%)
APRI score	0.82 ± 0.59	0.89 ± 0.78	0.78 ± 0.58

For categorical variables n (%) are presented. For continuous variables Mean (SD) or Median (Min;Max) are presented. BMI, body mass index; HCV, hepatitis C virus; ALT alaninaminotransferase, IP-10 interferon-γ inducible protein 10.

### Impact on HCV RNA

**Fig 2:**
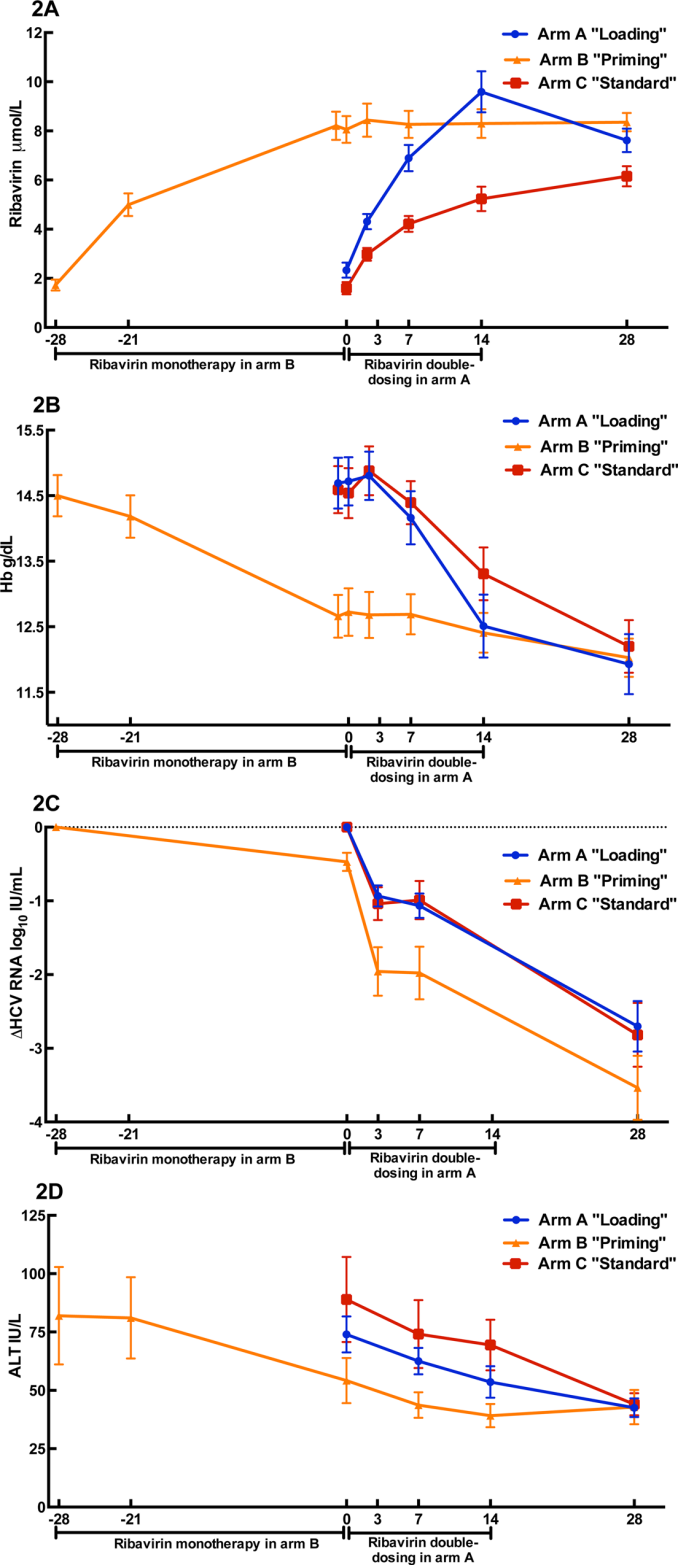
**Impact of “loading” (2 weeks of ribavirin double dosing concomitant with pegIFN-α), “priming” (4 weeks ribavirin mono-therapy prior to adding pegIFN-α), and “standard-of-care” on plasma ribavirin concentrations (A), hemoglobin (B), decline in HCV RNA (C), and ALT (D).** Mean with standard error of the mean shown.

**Fig 3:**
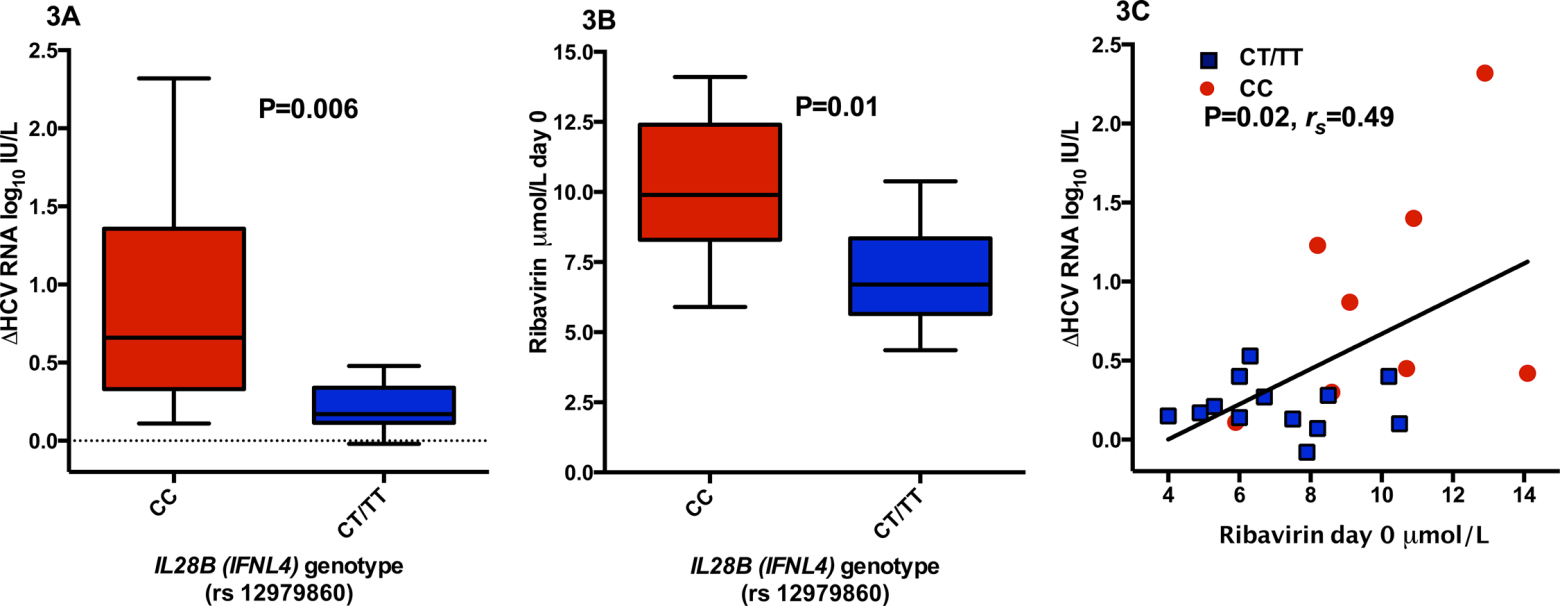
**Impact in arm B “priming” (i.e. 4 weeks ribavirin mono-therapy prior to adding pegIFN-α) of interleukin 28B (*IL28B*, also known as *IFNL4*) genetic variant CC (n = 8) vs. CT/TT (n = 13) on decline in HCV RNA (A) and ribavirin concentration day 0 (B), as well as correlation between decline in HCV RNA and ribavirin concentration day 0 (C).** White squares showing *IL28B* CC patients and black dots showing CT/TT carriage. Mean with standard deviation in (A and B). P values obtained using Mann-Whitney U test/Welch T test (3A), Mann-Whitney U test (3B) and Spearman correlation (3C).

In treatment arm B, patient were treated with weight-based ribavirin (13 mg/kg) mono-therapy for four weeks preceding the addition of pegIFN-α. During this period mean HCV RNA level decreased by 0.46 log_10_ IU/mL, from 5.83 (day -28) to 5.36 (day 0; P<0.0001 Wilcoxon matched pairs signed rank test) (Fig 2). The viral load reduction was significantly associated with *IL28B* genotype (0.89 vs. 0.21 log_10_ IU/mL, for CC (n = 8) and CT/TT (n = 13) respectively; P = 0.006, Mann-Whitney U test) (Fig 3), but not with pre-treatment plasma IP-10 concentrations. A positive correlation between the ribavirin concentration at day 0 in arm B “priming”, i.e. after four weeks of ribavirin mono-therapy, and viral decline was noted (P = 0.02, *r*_*s*_ = 0.49, Spearman correlation; Fig 3). Surprisingly in this study arm, carriage of the *IL28B* CC genetic variant entailed a significantly higher mean ribavirin concentration at day 0 compared to CT/TT (mean and standard deviation 10.1 ± 2.7 vs. 7.1 ± 2.0 μmol/L for CC and CT/TT respectively; P = 0.01, Mann-Whitney U test); however, when a stepwise linear regression model for decline in HCV RNA was performed including both ribavirin concentration day 0 and *IL28B* genotype, *IL28B* genetic variant was the only significant predictive variable (P = 0.004).

### Impact on ALT

During the four weeks of ribavirin mono-therapy, mean ALT decreased from 82 IU/L at baseline to 54 IU/L (P = 0.002, Wilcoxon signed rank test; Fig 2), with a mean fold decline of 0.71; 4 of 21 patients had ALT values within normal range at treatment initiation and 7 of 21 at day 0. A statistically significant association was noted between ALT and IP-10 fold change (P = 0.014, *r*_*s*_ = 0.53, Spearman correlation test), and a similar non-significant trend between ALT and HCV RNA reduction was observed (P = 0.055, *r*_*s*_ = -0.42). No significant association between *IL28B* genotype and ALT fold change was noted.

### Impact on IP-10 (CXCL10)

**Fig 4:**
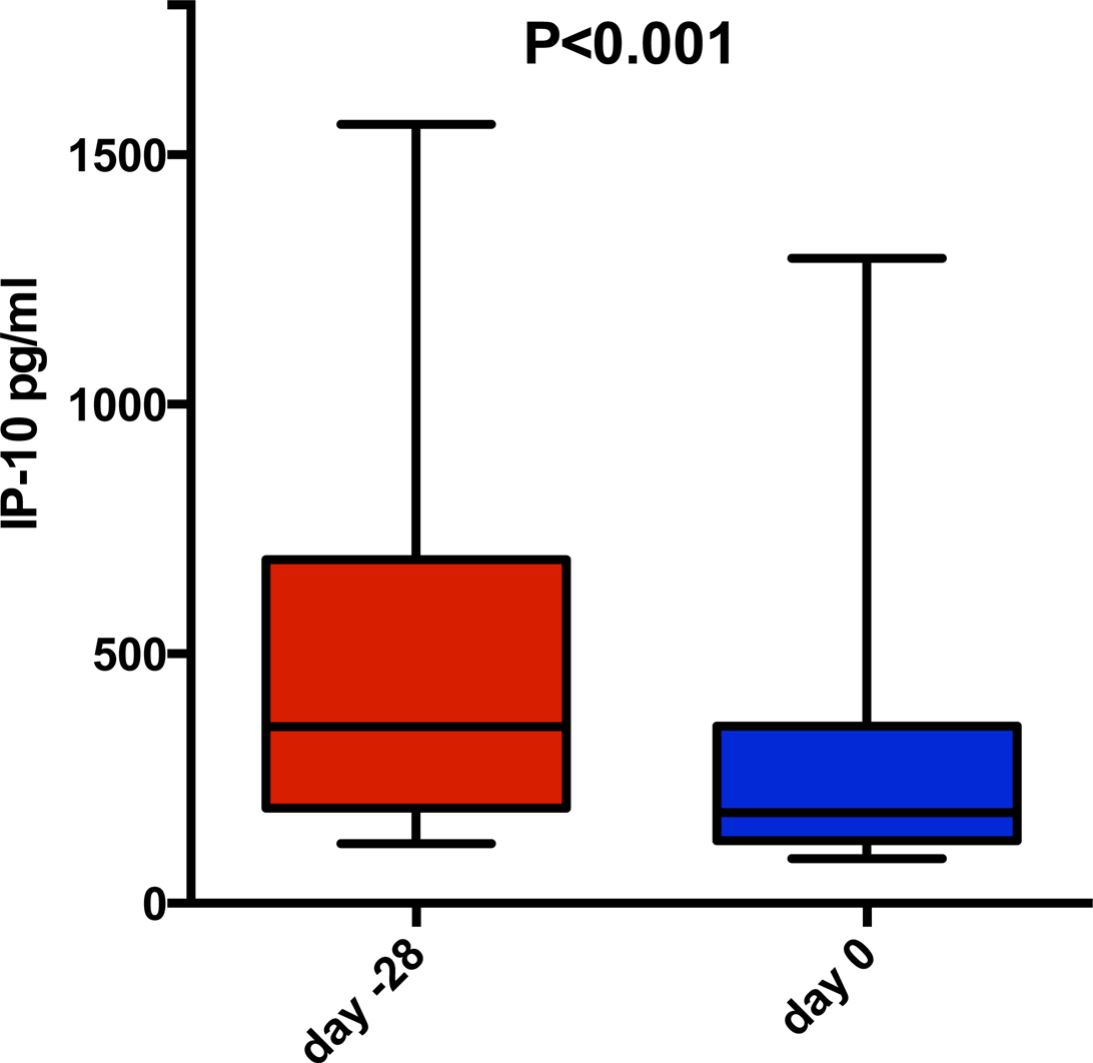
Impact of ribavirin mono-therapy for four weeks on plasma IP-10 concentrations. Box plots displaying the 10^th^, 25^th^, 50^th^, 75^th^, and 90^th^ percentiles. P values obtained using Wilcoxon matched-pairs signed rank test.

Interestingly, 4 weeks of ribavirin mono-therapy also was significantly associated with reduced mean IP-10 concentration from 548 to 345 pg/mL (P<0.001, Wilcoxon matched pairs signed rank test; Fig 4), and a significant reduction could be seen as early as after 1 week of therapy (548 to 466 pg/mL, P = 0.003). Interestingly in contrast to reduction in HCV RNA, no association between *IL28B* genotype and IP-10 decline was observed, similar to ALT. At baseline, plasma IP-10 and HCV RNA levels were significantly associated (P = 0.005, *r*_*s*_ = 0.59 Spearman correlation test), and a similar non-significant trend was observed between IP-10 fold reduction and HCV RNA decline during 4 weeks of ribavirin mono-therapy (P = 0.051, *r*_*s*_ = -0.43). Decline in IP-10 was not associated with ribavirin concentration after 4 weeks of mono-therapy (P = 0.47). Upon the initiation of pegIFN-α therapy, a rapid and similar increase in plasma IP-10 concentration was noted in all three arms (mean 6.9, 7.2, and 8.3 fold in arm A, B and C respectively; P = 0.73 Kruskall Wallis test), as has been reported previously from other studies [42, 43, 47], likely secondary to a systematic activation of interferon stimulated genes leading to a release of IP-10 from many cellular sources aside from HCV infected hepatocytes.

### Impact on ribavirin plasma concentrations

The “loading” arm, i.e. an initial 2-week double-dosage of ribavirin (i.e. 26 mg/kg/day) concomitant with pegIFN-α, entailed significantly higher mean plasma ribavirin concentrations compared to SOC (i.e. 13 mg ribavirin dosing/kg/day) at day 3 (4.3 vs. 3.0 μmol/L for “loading” and SOC respectively; P = 0.002, Mann-Whitney U test; Fig 2), day 7 (6.9 vs. 4.2 μmol/L; P<0.0001), and day 14 (9.6 vs. 5.2 μmol/L; P<0.0001). After 14 days of ribavirin double dosing in the “loading” arm A, ribavirin was reduced to standard dosing but at day 28 a minor, albeit significant difference in ribavirin concentration could still be observed (7.6 vs. 6.2 μmol/L for “loading” and SOC respectively; P = 0.04, Mann-Whitney U test). In the “priming” group, ribavirin concentrations reached steady state after 4 weeks of mono-therapy.

### Impact on hemoglobin

Patients in the “loading” arm suffered from a more pronounced mean hemoglobin decline from day 0 to day 14 compared to control patients receiving SOC (-2.18 vs. -1.38 g/dL; P = 0.03, Mann-Whitney U test; Fig 2). The difference in hemoglobin decline diminished after day 14, when normal ribavirin dosing was also administered in the “loading” arm. Apart from the planned dose reduction at day 14 in the “loading” arm, only one patient required a ribavirin dose adjustment during the first 28 days of therapy. This patient was enrolled in the “loading” arm and suffered from severe anemia, and ribavirin was discontinued after 14 days and all therapy was terminated after an additional 8 days. During the first 28 days, three patients in the “loading” arm and two patients in the SOC arm had a hemoglobin concentration below 10 g/dL. Interestingly, no patients in the “priming” group had a hemoglobin level below 10 g/dL during the first 4-weeks when ribavirin mono-therapy was administered, and only one patient during the first 28 days after the addition of pegIFN-α.

### Impact on outcome

**Fig 5:**
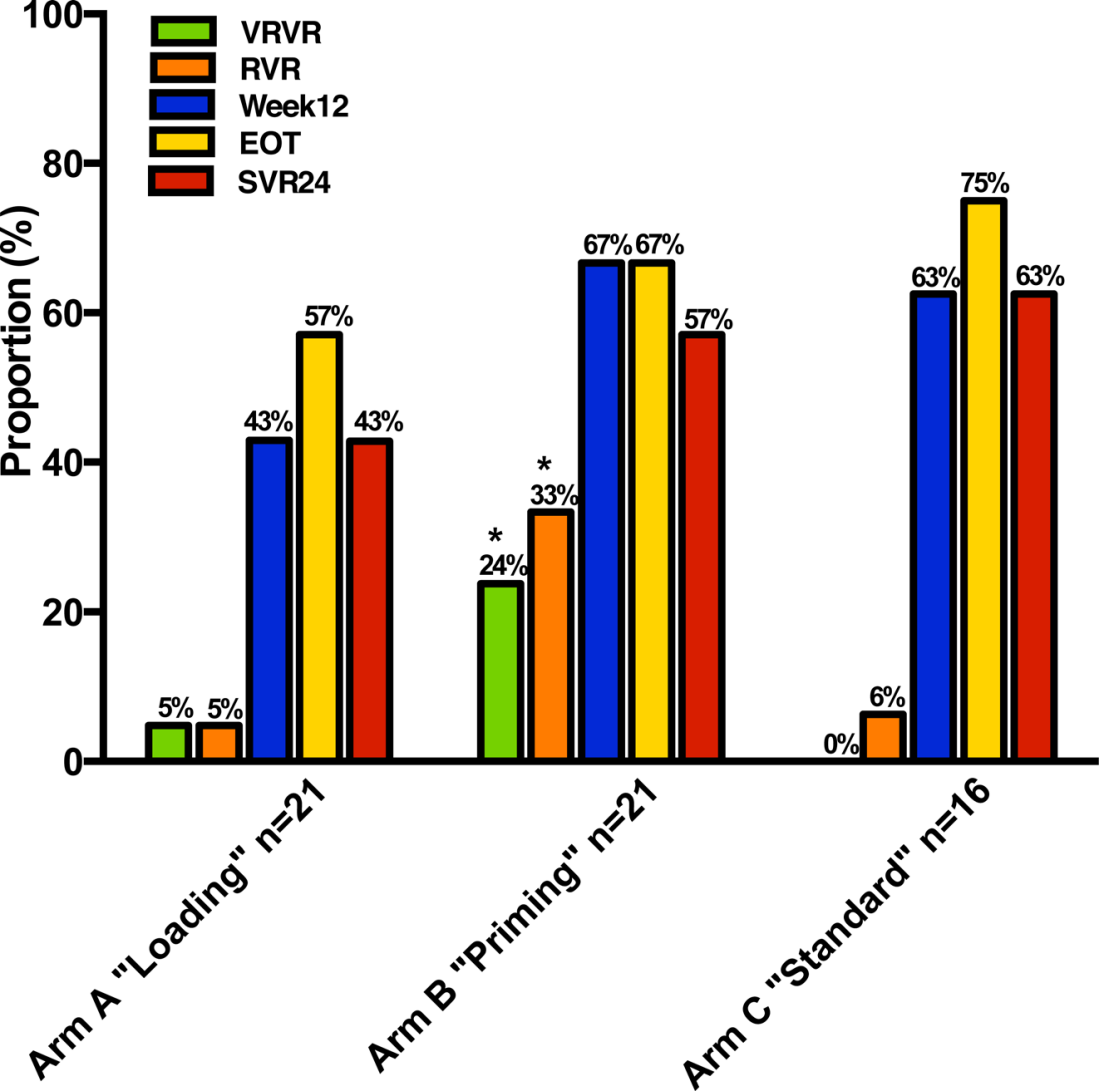
Proportion of patients achieving HCV RNA below 1000 IU/mL day 7 (VRVR), undetectable HCV RNA at week 4 (RVR), week 12 (Week 12), at end-of-treatment (EOT), and 24 weeks post treatment (SVR24).

No statistical significant differences between the experimental arms and the SOC arm regarding the first phase decline (mean and standard deviation 0.93 ± 0.66, 1.46, ± 0.89 and 1.04 ± 0.86 log_10_ IU/mL for arm A, B and C respectively; Fig 2) or the second phase decline (mean and standard deviation 1.64 ± 0.93, 1.53, ± 1.23 and 1.83 ± 0.83 log_10_ IU/mL for arm A, B and C respectively; Fig 2), which were the primary study endpoints, were noted. However, significantly more patients in the ribavirin mono-therapy”priming” arm achieved a very rapid virologic response (VRVR), i.e. HCV RNA below 1000 IU/mL day 7 (P = 0.02 for B vs. A+C, Fischer’s exact test; Fig 5), and this group maintained an approximately 1 log_10_ IU/mL greater decline in HCV RNA throughout the first 4 weeks after initiation of pegIFN-α compared to the other two groups (Fig 2). Similarly more patients in this study arm received 24 rather than 48 weeks of combination therapy, in accordance with the protocol, after achieving a rapid virological response (RVR), i.e. undetectable HCV RNA 4 weeks after initiation of pegIFN-α, as measured by their local laboratory (7 in “priming”, 2 in “loading”, and 1 in “SOC”). Nine of these ten patients treated for 24 weeks with combination therapy subsequently achieved SVR. Upon later evaluation at the central laboratory, significantly more patients in the ribavirin mono-therapy”priming” arm achieved RVR (7 in “priming”, 1 in “loading”, and 1 in “SOC”; P = 0.008 for B vs. A+C, Fischer’s exact test), and all achieving RVR as determined by the central laboratory subsequently achieved SVR, with the exception of one patient who terminated therapy after week 12 because of insomnia and was subsequently lost to follow-up; differences in the proportions achieving VRVR and RVR were predefined secondary study endpoints.

Interestingly *IL28B* genetic variant impacted on outcome, even in the ribavirin mono-therapy”priming” arm. Among the patients in the ribavirin mono-therapy”priming” arm achieving VRVR, 4 were *IL28B* CC carriers and 1 was CT, and among those achieving RVR, 6 were *IL28B* CC carriers and 1 was CT. Thus the proportion of CC_*rs12979860*_ as compared to non-CC was significantly higher among patients in the “priming” arm that achieved RVR as compared with those that did not (P = 0.0032; Fischer’s exact test). Among the patients in the “loading” arm, only one (*IL28B* CC carrier) achieved VRVR and RVR, and similarly in the “SOC” arm, only one (*IL28B* CC carrier) achieved RVR. Thus among *IL28B* CC carriers, patients in the ribavirin mono-therapy”priming” arm were more likely to achieve both VRVR (4 of 8 vs. 1 of 14 for priming vs. loading/SOC; p = 0.04, Fischer’s exact test) and RVR (6 of 8 vs. 2 of 14 for priming vs. loading/SOC; p = 0.008) as compared patients in the “loading” or “SOC” arms. In spite of differences in likelihood of achieving RVR, and thus total treatment duration, there were no significant differences in later on-treatment responses or SVR between the three study arms (Fig 5).

Whether or not patients had ribavirin concentrations above or below the pre-defined target concentration of 10.25 μmol/mL (or 2.5 mg/L), or above or below the median levels day 28, week 12, or end-of-treatment did not impact on the likelihood of achieving SVR. Similarly the mean ribavirin concentrations did not differ significantly day 28, week 12, or end-of-treatment between patients achieving or not achieving SVR (data not shown). As of 28 days after the initiation of pegIFN-α therapy, local assessments of ribavirin concentration were permitted. In the “loading” arm, this was performed in 13 patients up until study day 80, and 9 of these patients subsequently had an increase in ribavirin dosing because of low concentrations. In the “priming” and control arms, one patient in each arm similarly had an increase in ribavirin dosing because of sub-optimal local ribavirin concentrations between 28 and 80 days after initiation of pegIFN-α.

## Discussion

Neither a 4-week “priming” with ribavirin mono-therapy nor 2-weeks double dosing had any significant impact on the primary study endpoints, i.e. first and second phase decline, or on SVR, consistent with several previous studies [37, 48], but contrasting with the study by Quiles-Pérez et al. that noted an effect on early viral kinetics [36]. The lack of impact particularly on the second phase decline in HCV RNA in the “loading” arm was surprising, but may have resulted from double ribavirin dosing only being given for the first 2 weeks in addition to the use of relatively high ribavirin doses in all study arms. Nevertheless, significantly more patients receiving 4-weeks of ribavirin mono-therapy achieved VRVR and RVR, both of which were predefined secondary study endpoints, resulting in a greater proportion of patients qualifying for shortened treatment duration from 48 to 24 weeks of combination therapy, thus sparing considerable side effects and cost. However, one should bear in mind that in addition to the decline in HCV RNA observed after 4 weeks of ribavirin mono-therapy, patients in the “priming” arm also had a slightly lower baseline viral load, which in combination likely impacted on the likelihood of achieving VRVR and RVR.

Presently ribavirin is recommended for several DAA-based treatments for HCV genotype 1–3 infection [13], and likely will remain a vital component of therapy, especially for retreatment following relapse after DAA-based therapy to prevent the selection and emergence of resistance, or when preexisting baseline resistance associated variants (RAVs) have been detected. NS5A RAVs often exist prior to any exposure to NS5A-inhibitor inclusive DAA regimens [49, 50], and these RAVs often are enriched and persist following failure to achieve SVR. As ribavirin, when given as mono-therapy or in combination with interferon, does not appear to select for emergence of ribavirin-resistant variants [36, 51], and as viral replication is a prerequisite for development of RAVs, the findings in our present pilot study potentially may have implication for DAA-based, interferon-free therapy. For example, a 4-week “priming” with ribavirin mono-therapy could pave the way by modestly reducing the viral load prior to the initiation of DAAs, and thus reducing the risk of enrichment or selection of RAVs and secondary adaptive mutations that improve viral fitness, possibly allowing for shorter treatment duration. Indeed SVR was achieved in 83% of treatment-naïve HCV genotype 1 infected patients with baseline NS5A RAVs conferring greater than 100-fold resistance enrolled in the ION-3 trial as compared to 95–100% SVR for the remaining patients without such RAVs entailing a high loss of potency when treated with ledipasvir and sofosbuvir for 8 weeks [52]. One might speculate that ribavirin “priming” or concomitant administration might have improved the likelihood of achieving SVR in the face of baseline NS5A RAVs in this latter study by modestly lowering HCV RNA levels. Additionally the findings in our present study may help explain the higher likelihood of achieving SVR after both 12 and 24 weeks of ledipasvir and sofosbuvir therapy for HCV genotype 1a infection when ribavirin was co-administered as compared to when ribavirin was not given in the face of baseline NS5A RAVs (88% without vs. 94% with ribavirin, and 85% without vs. 100% with ribavirin for 12 and 24 weeks of therapy respectively) [53].

In addition to shorter overall treatment duration among patients receiving 4-weeks “priming” with ribavirin mono-therapy, several other intriguing observations were made in the present study. Four weeks of ribavirin mono-therapy resulted in a mean HCV RNA decline of 0.46 log_10_ IU/mL, which is consistent with previous studies [23, 37, 48]. However, it also was noted that the viral decline was significantly associated with *IL28B* genotype, with CC carriage entailing a greater viral decline as compared to CT/TT. Interestingly, in the study by Rotman et al. a similar, non-significant trend was observed towards slightly greater decline in HCV RNA among *IL28B* CC/CT carriers as compared to *IL28B* TT receiving ribavirin mono-therapy, as well as a significantly greater decline in ALT among *IL28B* CC patients [48]. In contrast, in the study by Mihm et al., no association was noted between HCV RNA and *IL28B* genetic variant, possible secondary to the limited sample size [37]. Similarly ribavirin concentrations after four weeks of mono-therapy were significantly associated with decline in HCV RNA, which also previously has been noted [23], but surprisingly also with *IL28B* genetic variation. In an attempt to further delineate the impact on clearance of HCV RNA, a stepwise linear regression model for viral decline was performed, including both *IL28B* polymorphism and ribavirin concentration. In this analysis only *IL28B* CC carriage remained a significant predictor of viral decline during the 4 weeks of ribavirin mono-therapy. Since genetic variants in proximity to *IL28B* previously have been associated with HCV RNA reduction during pegIFN-α and ribavirin combination therapy [39], spontaneous resolution of HCV infection [40, 54] and recently also to early viral kinetics and treatment outcome in interferon-free HCV therapeutic trial [54, 55], it is reasonable that an impact also could be present during ribavirin mono-therapy. It is important to bear in mind that during ribavirin mono-therapy, endogenously produced interferon is present in spite of the lack of exogenous administration, and that the addition of ribavirin may trigger further *IL28B* associated innate anti-viral interferon responses, either by directly lowering the viral load or indirectly by modifying host gene expression [24–27, 56].

Ribavirin mono-therapy also decreased the plasma concentration of IP-10, which has been reported previously [48]. In this study there was a significant association between baseline plasma IP-10 and HCV RNA, and there also was a trend towards an association between IP-10 fold change and reduction in HCV RNA after 4 weeks of mono-therapy. A similar relationship has been noted in studies using ribavirin mono-therapy or interferon-free HCV therapy [48, 57, 58]. Thus a possible explanation is that the decline in IP-10 is driven by the decline in viral load, although the decline in HCV RNA during ribavirin mono-therapy was regulated by *IL28B* genetic variation, which the decline in IP-10 was not. Similarly, in the study by Meissner et al., HCV RNA kinetics and IP-10 decline were significantly correlated among 60 treatment-naïve HCV genotype 1 infected patients receiving ribavirin and sofosbuvir for 24 weeks, but in spite of undetectable HCV RNA in all end-of-treatment samples, IP-10 levels were somewhat elevated in the 14 patients who later relapsed, also suggesting that HCV RNA levels do not exclusively regulate IP-10 [59]. A similarly plausible, alternative molecular mechanism is a ribavirin-powered modulation of ISGs, including IP-10, in congruence with prior *in vivo* [25] and *in vitro* [26, 27] studies, and lower baseline induction of ISGs has been reported to be associated with favorable outcome following interferon-based HCV therapy [60]. The findings in the ribavirin mono-therapy “priming” arm in the present study that the decline in both IP-10 and ALT, unlike the reduction in HCV RNA, was not associated with host *IL28B* genetic variation may further support this latter hypothesized mechanism of action of ribavirin.

As expected ribavirin “loading” with ribavirin double-dose (26 mg/kg/day) for two weeks resulted in more rapid increase in ribavirin concentration as compared to standard dosing (13 mg/kg/day). Ribavirin concentration reportedly is most important early during combination therapy, but higher ribavirin concentrations also increase the risk of anemia [61, 62]. In this study the higher ribavirin concentration led to faster decline in hemoglobin from day 0 to day 14, and to a ribavirin treatment termination in one patient, but did not impact outcome. Interestingly, patients in the double-dosage, “loading” arm had achieved high ribavirin concentrations already by day 7 in parity with levels achieved after 28 days among control patients receiving standard-of-care dosing, without any major impact on hemoglobin concentrations. Similarly by day 14, patients in the “loading” arm reached peak ribavirin concentrations, and levels subsequently decreased upon reverting to standard ribavirin dosing, i.e. 13 mg/kg/day. Thus it appears that 14 days of “loading” might have been excessive, and that in severe viral infections, where it is crucial to rapidly achieve high concentrations, one-week of ribavirin double dosing may be preferential.

The present pilot study, similar to previously published studies exploring alternative dosing of ribavirin for HCV [36, 37, 48], suffered from insufficient power to detect minor differences in SVR, which obviously is a limitation as small sizes risk introducing biases in addition to hampering the detection of significant findings. Our primary endpoint thus was focused on early on-treatment viral kinetics, as ribavirin reportedly predominantly impacts on the second phase decrease in HCV RNA [63], and the initial power calculation performed was based on the recruitment of 35 patients in each arm, a number we were unable to recruit, likely secondary to the rapid introduction of DAA-based HCV therapy. In spite of this, many significant and important findings were made in the present study, which may further knowledge in the field.

Thus in conclusion, this pilot study indicates that (i) ribavirin mono-therapy may have an anti-viral effect differently regulated across *IL28B* genotypes, entails higher likelihood of achieving VRVR as well as RVR and thus of shortened treatment duration, and down-regulates IP-10 independent of *IL28B*, and (ii) one-week of ribavirin double dosing may be sufficient to achieve high concentrations.

## Supporting Information

S1 File The study protocol for the RibaC trial. (DOC)

S2 File The CONSORT checklist for the RibaC trial. (DOC)
