# Supplementary material for: Randomized Trial Evaluating the Impact of Ribavirin Mono-Therapy and Double Dosing on Viral Kinetics, Ribavirin Pharmacokinetics and Anemia in Hepatitis C Virus Genotype 1 Infection
Source: PLoS One. 2016 May 11;11(5):e0155142. doi: 10.1371/journal.pone.0155142 (PMC4864304; doi:10.1371/journal.pone.0155142)
Supplement: S1 File — (DOC) [file pone.0155142.s001.doc]

A Randomized, Open-label, Parallel Group, Multicenter Pilot Study Evaluating the Efficacy and Safety of Alternative Dosing of Ribavirin vs. Standard of Care Dosing in Combination with Peginterferon alpha-2a in Interferon Naïve Patients with Chronic Hepatitis C Genotype 1 Infection

An Investigator Initiated Study

*RibaC*

**Study Steering Committee**

Chairman: Martin Lagging

Co-Chairman: Johan Westin

Sweden: Karin Lindahl, Gunnar Norkrans

Denmark: Peer Christensen, Mads Rauning Buhl

Finland: Martti Färkkilä

Norway: Olav Dalgard

**ADMINISTRATIVE AND CONTACT INFORMATION**

**Steering Committee Members:**

Martin Lagging MD, PhD., Assoc. Prof. Johan Westin MD, PhD, Assoc. Prof.

Dept. of Infectious Diseases/Virology Dept. of Infectious Diseases/Virology

University of Gotheburg University of Gotheburg

Guldhedsgatan 10B Guldhedsgatan 10B

SE-413 46 Göteborg SE-413 46 Göteborg

Phone: +46 31 342 46 58 Phone: +46 31 342 46 58

Mobile: +46 705 68 37 59 Mobile: +46 708 56 92 82

Fax: +46 31 411 256 Fax: +46 31 411 256

epost: [martin.lagging@medfak.gu.se](mailto:martin.lagging@medfak.gu.se) johan.westin@gu.se

SWEDEN:

Karin Lindahl MD, PhD

Infektionskliniken

Karolinska Universitetssjukhuset/Huddinge

14186 Stockholm

epost: [karin.lindahl@ki.se](mailto:karin.lindahl@ki.se)

Gunnar Norkrans MD, PhD, Prof.

Dept of Infectious Diseases
Sahlgrenska University Hospital/Östra
SE-416 85 GÖTEBORG
Phone +46 (0)31 3434248
Fax   +46 (0)31 847813

epost: [gunnar.norkrans@vgregion.se](mailto:gunnar.norkrans@vgregion.se)

DENMARK:

Peer Christensen MD, PhD, Assoc. Prof.

Dept. Of Infectious Diseases, Odense University Hospital

University of Southern Denmark

Sdr. Boulevard 29

DK-5000 Odense C

Phone: +45 6541 3845

Mobile: +45 2860 1206

Fax: +45 6611 7418

Epost: peer.christensen@dadlnet.dk

Mads Rauning Buhl
Infektionsmedicinsk Afdeling Q
Skejby Sygehus
Brendstrupgårdsvej 100
8200 Århus N
Email: madsbuhl@rm.dk
tlf  89 49 83 03
Fax: 89 49 83 10

FINLAND:

Martti Färkkilä

Helsinki University Central Hospital

Clinic of Gastroenterology

BO 340

00029 HUS

tel +358504271622

fax +358947174688

epost: martti.farkkila@hus.fi

NORWAY:

Olav Dalgard, MD, PhD

Dept. of Medicine

Oslo University Hospital Rikshospitalet

NO-0027 Oslo

Phone: +47 23070000

Mobile: +33 6 79777464

**ADMINISTRATIVE AND CONTACT INFORMATION (CONT.)**

Gothia Forum

Medicinaregatan 8 B

413 90 Göteborg

Sweden
**Telefon:** 031 - 342 70 96
**Mobil:** 0708 - 76 01 30

**Data Management and Biostatistics Group (CRO):**

Gothia Forum

Medicinaregatan 8 B

413 90 Göteborg

Sweden
**Telefon:** 031 - 342 70 96
**Mobil:** 0708 - 76 01 30

SYNOPSIS OF PROTOCOL: RibaC

(**Riba**virin Loading Dose or Priming and Concentration Targeting for H**C**V Genotype 1)

| TITLE | A Randomized, Open-label, Parallel Group, Multicenter Pilot Study Evaluating the Efficacy and Safety of Alternative Dosing of Ribavirin vs. Standard of Care Dosing in Combination with Peginterferon alpha-2a in Interferon Naïve Patients with Chronic Hepatitis C Genotype 1 Infection | | |
| --- | --- | --- | --- |
| STEERING COMMITTEE | Chairman: Martin Lagging  Co-Chairman: Johan Westin  Denmark: Peer Christensen, Mads Rauning Buhl  Finland: Martti Färkkilä  Norway: Olav Dalgard  Sweden: Karin Lindahl, Gunnar Norkrans | CLINICAL PHASE | III |
| INDICATION | Chronic hepatitis C (CHC) genotype 1 infection | | |
| OBJECTIVES | Primary   - To demonstrate the efficacy of (A) 2 weeks of high dose of ribavirin (“loading”, ≥26 mg/kg/day for 14 days followed by ≥13 mg/kg/day) followed by concentration targeted (≥ 2.5 mg/L (10.25 μmol/L) 28 days after initiation of ribavirin therapy) dosing of ribavirin vs. (B) 4 weeks of ribavirin dosing before initiation of PEG-interferon dosing (“priming”, ≥13 mg/kg/day) followed by concentration targeted (≥ 2.5 mg/L (10.25 μmol/L) 28 days after initiation of ribavirin therapy) dosing of ribavirin in combination with peginterferon alpha-2a in interferon naïve patients with chronic hepatitis C (CHC) virus genotype 1 infection as compared to (C) standard-of-care dosing of ribavirin (≥13 mg/kg/day without monitoring of ribavirin concentrations) in combination with peginterferon alpha-2a as evaluated by the early viral kinetic response measured by effect on the initial decline of HCV-RNA (during the first days after initiating peginterferon and ribavirin therapy) and second phase decline (day 7 to week 12 of therapy).   Secondary  To prospectively evaluate:   - the efficacy of 2 week loading or 4 week priming followed by concentration targeted dosing of ribavirin in combination with peginterferon alpha-2a in interferon naïve patients with chronic hepatitis C (CHC) virus genotype 1 infection as compared to standard-of-care dosing of ribavirin in combination with peginterferon alpha-2a as evaluated by the proportion of patients achieving VRVR ( “very rapid virologic response” i.e. HCV-RNA below 1000 U/mL at treatment day 7 after intiation of PEG-interferon), RVR (“rapid virologic response”, undetectable HCV-RNA at treatment day 28 after intiation of PEG-interferon), cEVR (“complete early virologic response”, i.e. undetectable HCV-RNA at treatment week 12 after intiation of PEG-interferon), and pEVR (“partial early virologic response”, i.e. decline of HCV-RNA by at least 2 log10 compared with baseline at treatment week 12 after intiation of PEG-interferon). - the efficacy of 2 week loading or 4 week priming followed by concentration targeted dosing of ribavirin in combination with peginterferon alpha-2a in interferon naïve patients with chronic hepatitis C (CHC) virus genotype 1 infection is superior to standard-of-care dosing of ribavirin, peginterferon alpha-2a therapy as evaluated by sustained virological response (SVR, undetectable HCV-RNA 24 weeks after end of treatment). - the predictive value of monitoring of viral load at day 0 (before the 1^st^ dose of peginterferon alpha-2a), day 3, day 7 (before the 2^nd^ dose of peginterferon alpha-2a), and day 28 (before the 5^th^ dose of peginterferon alpha-2a) determining which patients will obtain a sustained virological response (SVR) when treated with ribavirin and peginterferon alpha-2a therapy. - the association between the trough concentrations of ribavirin (day 1, day 3, day 7, day 14, day 28, week 8, week 12, week 18, and end-of-treatment), and the therapeutic efficacy of ribavirin and peginterferon alpha-2a therapy. - The association between plasma IP-10 (days 0, 1, 3, 7, and 14, and weeks 8, 18, and 24), and the therapeutic efficacy of ribavirin and peginterferon alpha-2a therapy. - the effect of IL-28B ploymorfism on the viral kinetic response. - the effect of baseline vitamin D concentrations on the viral kinetic response. - the effect of baseline IP-10 concentrations on the viral kinetic response. - the association between liver histology as evaluated by the Ishak scoring system (modified HAI score) and steatosis grading if a liver biopsy has been performed, and the therapeutic efficacy of peginterferon alfa-2 combination therapy with ribavirin. - the association between liver stiffness as evaluated by the FibroScan if this has been performed and the therapeutic efficacy of peginterferon alfa-2 combination therapy with ribavirin. - the association between liver fibrosis as evaluated by the GUCI and APRI indexes as well as hyaluronic acid, and the therapeutic efficacy of peginterferon alfa-2 combination therapy with ribavirin. - the association between body mass index (BMI), waist circumference, weight and age, and the therapeutic efficacy of peginterferon alfa-2 combination therapy with ribavirin. - the predictive value of monitoring CD56 negative NK, CD4^+^ CD38^+^, CD8^+^ CD38^+^, CD8^+^CD16^+^, Foxp3^+^, and pDC (CD303^+^) cells in peripheral blood and liver biopsies as analyzed by FACS prior to and after the initiation of therapy for determining which patients will obtain a sustained virological response (SVR) when treated with peginterferon alfa-2 and ribavirin combination therapy. - the predictive value of quantifying mRNA expression in pre-treatment liver biopsies for IFI-27, IP-10, CXXL-6, IL-2, IL-8, IL-10, γ-IFN, TNF-α, KRT19, COL1A1, MMP7, TIMP1, FASL, AIF1 for determining which patients will obtain a sustained virological response (SVR) when treated with peginterferon alfa-2 and ribavirin combination therapy.   Others:  To prospectively evaluate:   - the predictive value of monitoring the trough concentrations of ribavirin (day 1, day 3, day 7), and the final steady-state concentration of ribavirin.. - the safety of peginterferon alpha-2a combination therapy with concentration targeted dosing of ribavirin in combination peginterferon alpha-2a, based on accumulated number of adverse events and severe adverse events as well as the impact on quality of life during treatment and follow-up phase. - the safety of peginterferon alpha-2a combination therapy with concentration targeted dosing of ribavirin in combination peginterferon alpha-2a, with particular regards to the number of patients in each treatment arm developing anemia grade 1 (10.5-9.5 g/dL), grade 2 (9.4-8.0 g/dL), grade 3 (7.9-6.5 g/dL), and grade 4 (<6.5 g/dL). | | |
| TRIAL DESIGN | Multicenter, randomized, open-label, parallel group pilot study.  Randomization to the three treatment arms A, B and C will be stratified by gender, age above or below 40 years, and presence of liver cirrhosis/non-cirrhosis (as evaluated by the Gothenburg University Cirrhosis Index (GUCI) ≥1.0). | | |
| DISCONTINUATION SCHEME | Treatment will be stopped in patients having a decline of HCV-RNA less than 2-log_10_ after 12 weeks or detectable HCV-RNA after 24 weeks of study treatment as measured by Roche COBAS AmpliPrep/COBAS TaqMan HCV Test (≤15 IU/mL) | | |
| NUMBER OF SUBJECTS | Total of 105 patients, 35 per treatment group. | | |
| TARGET POPULATION | Men and women >18 years old with CHC genotype 1 will be enrolled in this study. Patients must have quantifiable HCV-RNA, and must have compensated liver disease (Child-Pugh Grade A). Liver biopsies and/or FibroScan evaluations are encouraged especially for patients with a Gothenburg University Cirrhosis Index (GUCI, a cirrhosis index calculated by (normalized AST x PK-INR x 100) / Platelet count (x 10^9^/L)) ≥1.0, but not mandatory. Patients with other forms of liver disease, human immunodeficiency virus (HIV) infection, hepatocellular carcinoma, anemia, pre-existing severe depression or other psychiatric disease, significant cardiac disease, renal disease, seizure disorders, or severe retinopathy, or previously treated with interferon/ peg-interferon with or without ribavirin are excluded. | | |
| SELECTION CRITERIA | Inclusion Criteria:   - Written informed consent - Male and female patients ≥18 years of age - Serologic evidence of chronic hepatitis C infection by an anti-HCV antibody test - Serum HCV-RNA ≥15 IU/mL. - HCV genotype 1 infection confirmed within the past 2 years preceding the initiation of test drug dosing. - Compensated liver disease (Child-Pugh Grade A clinical classification) - Patients with cirrhosis or transition to cirrhosis must have an abdominal ultrasound, CT scan, or MRI scan without evidence of hepatocellular carcinoma and a serum AFP ≤100 ng/mL within 2 months of randomization - Negative urine or blood pregnancy test (for women of childbearing potential) documented within the 24-hour period prior to the first dose of study drug - All fertile males and females receiving ribavirin must be using effective contraception during treatment and during 4 months for female patients / 7 months for male patients after end of treatment - Subject must weigh between 45 and 105 kg at screening   Exclusion Criteria:   - Women with ongoing pregnancy or breast feeding - IFN/ peg-interferon with or without ribavirin therapy at any previous time - Therapy with any systemic anti-viral, anti-neoplastic or immunomodulatory treatment (including supraphysiologic doses of steroids and radiation) ≤6 months prior to the first dose of study drug - Any investigational drug ≤6 weeks prior to the first dose of study drug. - HCV genotype 2, 3, 4, 5, 6, or 7 infection. - Positive test at screening for anti-HAV IgM Ab, HBsAg, anti-HBc IgM Ab, anti-HIV Ab - Evidence of a medical condition associated with chronic liver disease other than HCV (e.g., hemochromatosis, autoimmune hepatitis, metabolic liver disease, alcoholic liver disease, toxin exposures) - History or other evidence of decompensated liver disease - Neutrophil count <1500 cells/mm^3^ or platelet count <90,000 cells/mm^3^ at screening - Serum creatinine level >2 mg/dl (>124 µmol/L) or creatinine clearance ≤50 ml/minute at screening - Severe psychiatric disease, especially depression, as judged by the treating physician. - History of a severe seizure disorder or current anticonvulsant use - History of immunologically mediated disease, severe chronic pulmonary disease associated with functional limitation, severe cardiac disease, major organ transplantation or other evidence of severe illness, malignancy, or any other conditions which would make the patient, in the opinion of the investigator, unsuitable for the study - Thyroid dysfunction not adequately controlled (TSH and T4 levels out of normal range) - Evidence of severe retinopathy (e.g. CMV retinitis, macula degeneration) or clinically relevant ophthalmological disorder due to diabetes mellitus or hypertension - Evidence of drug abuse (including excessive alcohol consumption) in accordance with local therapeutic traditions. - Inability or unwillingness to provide informed consent or abide by the requirements of the study - Male partners of women who are pregnant - Hemoglobin <12 g/dL in women or <13 g/dL in men at screening. - Any patient with an increased baseline risk for anemia (e.g. thalassemia major, spherocytosis, history of GI bleeding, etc) or for whom anemia would be medically problematic coagulopathia. - Patients with documented or presumed coronary artery disease or cerebrovascular disease should not be enrolled if, in the judgment of the investigator, an acute decrease in hemoglobin by up to 4 g/dL (as may be seen with ribavirin therapy) would not be well-tolerated - Evidence of allergy to PEG-IFN or ribavirin. | | |
| LENGTH OF STUDY | Period of enrollment: 52 weeks | | |
| Group A (“Loading”): PEG-IFN α-2a 180 μg/week plus loading (≥26 mg/kg/day for 2 weeks followed by ≥13 mg/kg/day) and concentration targeted (≥ 2.5 mg/L, i.e ≥ 10.25 μmol/L, as measured after 4 weeks of therapy) dosing of ribavirin and response guided treatment duration (RVR 24 weeks, non-RVR 48 weeks, pEVR consider 72 weeks), follow-up period 24 weeks | | |
| Group B (“Priming”): Standard-of-care dosing of ribavirin (≥13 mg/kg/day) without PEG-IFN for 4 weeks followed by 24-48 additional weeks of PEG-IFN α-2a 180 μg/week plus standard-of-care dosing of ribavirin (≥13 mg/kg/day) and concentration targeted (≥ 2.5 mg/L, i.e ≥ 10.25 μmol/L, as measured after 28 days after the initiation of ribavirin) dosing of ribavirin and response guided treatment duration (RVR 28 weeks, non-RVR 52 weeks, pEVR consider 76 weeks), follow-up period 24 weeks  Group C (“Standard-of-Care”): PEG-IFN α-2a 180 μg/week plus standard-of-care dosing of ribavirin (≥13 mg/kg/day without any measurement of ribavirin concentration) and response guided treatment duration (RVR 24 weeks, non-RVR 48 weeks, pEVR consider 72 weeks), follow-up period 24 weeks | | |
| DURATION OF STUDY | October 2010 till July 2013 including a recruitment period of 52 weeks, with an interim analysis of the primary study endpoints after all patients reached treatment 12 week. | | |
| INVESTIGATIONAL PRODUCT(S) DOSE/ ROUTE/ REGIMEN | Peginterferon α-2a  Copegus (ribavirin): 200-400 mg/tablet | | |
| ASSESSMENTS OF: |  | | |
| - EFFICACY | Primary Variable:   - The early virological response as measured by decline in HCV-RNA during the first 12 weeks of peginterferon alpha-2a and ribavirin therapy in the three study arms.   Secondary Variables:   - VRVR, RVR, cEVR, and pEVR rates defined as percentage of patients achieving these goals as measured by TaqMan PCR in the three study arms. - Percentage of patients with non-detectable HCV-RNA at study day 1, 3, 7, 14, 21, 28, week 8, week 12, end of treatment, and 24 weeks after completion of treatment as measured by TaqMan PCR as compared to day 0. - SVR rate defined as percentage of patients with non-detectable HCV-RNA as measured by TaqMan PCR at 24 weeks post completion of the treatment period in the three study arms. - Relapse rates defined as percentage of patients with non-detectable HCV-RNA as measured by TaqMan PCR at the end-of-treatment but with detectable HCV-RNA 24 weeks post completion of the treatment period in the three study arms. - SVR rate and percentage of patients with normal serum ALT levels at 24 weeks after completion of the treatment period in the total patient population, and its association with the following factors: - early virological response - pharmacokinetic response to ribavirin | | |
| - SAFETY | - Adverse event rate and profile - Laboratory assessments - An independent DSMB will evaluate safety after the first 30 patients have reached treatment weeks 2 and 12. - The use of erythropoietin (NeoRecormon) ≥3000 IE 3 times week sc initially in the event of anemia is permitted throughout the study at the discretion of the treating physician with the exception if the patient has any of the following exclusion criteria for the use of erythropoietin: platelet count >500,000, risk of thrombosis, or poorly controlled hypertension, especially if hemoglobin decreases persistently to < 10.0 g/dL or in the event of a rapid decrease in hemoglobin exceeding 4.0 g/dL over a 2 week period. If erythropoietin is initiated, a new ribavirin concentration at local lab should be evaluated after 4 weeks in Groups A (“Loading”) and B (“Priming”). - In the event of anemia, dose reductions of ribavirin are permitted in all study arms throughout the study at the discretion of the treating physician. - If hemoglobin decreases to < 8.0 g/dL, ribavirin should be discontinued. - If hemoglobin decreases to < 7.0 g/dL, blood transfusion should be considered at the discretion of the treating physician. - If hemoglobin decreases to < 6.5 g/dL, blood transfusion should be given. - Patients will discontinue therapy if the decrease in plasma HCV RNA between week 12 and baseline is ≤ 2 log_10_ IU/mL or if plasma HCV RNA is still detectable by treatment week 24. - The use of granulocyte growth factors are permitted throughout the study in accordance with local or national treatment guidelines at the discretion of the treating physician. - The members of the Steering Committee in each respective country will be responsible for monitoring the participating centers in their country. One or more experienced monitor is recommended per participating country. - Only experienced treatment centers willing to include ≥ 5 patients will be permitted to participate in the study. - All serious adverse events in the study will be reported to IST and Welwyn. | | |
| - QUALITY OF LIFE | - SF 36 | | |
| STATISTICAL ANALYSES | Sample Size Calculation: This is a pilot trial with the viral kinetic response measured as described above, during the first 12 weeks of therapy as primary endpoint with the aim of investigating the feasibility and safety of “loading” and “priming” dosing of ribavirin in conjunction with PEG-IFN for chronic HCV genotyp 1. To demonstrate an increase in the reduction of HCV RNA from day 0 to 3 from 0.9 log_10_ IU/mL in Group C (Standard-of-Care) to 1.4 log_10_ IU/mL in Group A or B or an increase in the reduction of HCV RNA from day 7 to 28 from 0.4 log_10_ IU/mL/week in Group C (Standard-of-Care) to 0.6 log_10_ IU/mL/week in Group A or B the study requires at least 35 patients per study arm. The statistical power (chance) for the study to detect a superior effect in Group A or B as compared to C is 80%. Statistical sample-size calculation is based on z-test for differences between proportions and is one-sided with a significance level of 5%.  Analysis Plan: Intention-to-treat and Per-protocol analysis on primary endpoints will be performed for patients receiving at least one dose of the study drugs. Additionally, an analysis of SVR with regards to whether or not the target concentration of ribavirin 2.5 mg/L (10.25 μmol/L) at day 28 was achieved across all study arms will be performed. | | |

TABLE OF CONTENTS..................................................................................................Page

Part I: Study design and conduct [1](#__RefHeading___Toc138151525)

1. background and rationale [1](#__RefHeading___Toc138151526)

1.1 Background [1](#__RefHeading___Toc138151527)

1.2 Study Medications [1](#__RefHeading___Toc138151528)

1.2.1 PEGASYS® (Peginterferon-alpha-2a, PEG-IFN 2a) [1](#__RefHeading___Toc138151529)

1.2.2 Copegus® (Ribavirin) [2](#__RefHeading___Toc138151530)

1.3 Clinical Experience [2](#__RefHeading___Toc138151531)

1.3.1 Combination therapy of Peginterferon-alpha-2a and Ribavirin [2](#__RefHeading___Toc138151532)

1.4 Rationale [2](#__RefHeading___Toc138151533)

1.4.1 Rationale for the Study Design [2](#__RefHeading___Toc138151534)

1.4.2 Viral kinetics [3](#__RefHeading___Toc138151535)

1.4.3 Liver Fibrosis and Inflammation Score [3](#__RefHeading___Toc138151536)

1.4.4 Rational for Dosage Selection [3](#__RefHeading___Toc138151537)

1.4.4.1 Pegasys [3](#__RefHeading___Toc138151538)

1.4.4.2 Ribavirin [4](#__RefHeading___Toc138151539)

2. OBJECTIVES OF The STUDY [4](#__RefHeading___Toc138151540)

2.1 Primary Objective [4](#__RefHeading___Toc138151541)

2.2 Secondary Objectives [4](#__RefHeading___Toc138151542)

3. STUDY design [6](#__RefHeading___Toc138151543)

3.1 Overview of Study Design and Dosing Regimen [6](#__RefHeading___Toc138151544)

3.2 Number of Patients [7](#__RefHeading___Toc138151545)

3.3 Centers [7](#__RefHeading___Toc138151546)

4. STUDY POPULATION [7](#__RefHeading___Toc138151547)

4.1 Target Population [7](#__RefHeading___Toc138151548)

4.2 Inclusion Criteria [7](#__RefHeading___Toc138151549)

4.3 Exclusion Criteria [8](#__RefHeading___Toc138151550)

4.4 Concomitant Medication and Treatment [9](#__RefHeading___Toc138151551)

5. SCHEDULE OF ASSESSMENTS AND PROCEDURES [9](#__RefHeading___Toc138151552)

5.1 Schedule of Assessments [9](#__RefHeading___Toc138151553)

5.2 Screening Examination and Eligibility Screening Form 13

5.3 Study Assessments 14

5.3.1 Efficacy Assessments 14

5.3.2 Safety Assessments 16

6. end points of the study 17

6.1 Primary Endpoint 17

6.2 Secondary Efficacy Endpoints 18

7. STUDY MEDICATIONS 18

7.1 Dose and Schedule of Study Medications 18

7.2 Preparation and Administration of Study Medication 19

7.3 Blinding and Randomization 19

7.4 Compliance 19

7.5 Treatment Duration 19

7.5 Stopping Rule 20

8. SAFETY ISSUES 20

8.1 Adverse Events and Laboratory Abnormalities 20

8.1.1 Clinical Adverse Events 20

8.1.1.1 Severity 20

8.1.1.2 Relationship 21

8.1.2 Laboratory Test Abnormalities 21

8.2 Handling of Safety Parameters 22

8.2.1 Serious Adverse Events (Immediately Reportable to Roche) 22

8.2.2 Treatment and Follow-up of Adverse Events 23

8.2.3 Follow-up of Abnormal Laboratory Test Values 23

8.2.4 Pregnancy 23

8.3 Dose Adjustment Guidelines for Intolerance 24

8.3.1 PEGASYS® Dose Modifications 24

8.3.1.1 General Dose Reduction Guidelines 25

8.3.2 Ribavirin Dose Modifications 25

8.4 Premature Withdrawal 26

8.5 Warnings and Precautions 27

8.5.1 Peginterferon-alpha-2a 27

8.5.2 Ribavirin 28

8.5.2.1 Pregnancy 28

8.5.2.2 Carcinogenesis and Mutagenesis: 29

8.5.2.3 Adverse Reaction 29

9. STATISTICAL considerations and ANALYtical plan 29

9.1 Primary and Secondary Study Variables 29

9.1.1 Primary Variable 29

9.1.2 Secondary Efficacy Variables 30

9.1.3 Secondary Safety Variables 30

9.2 Statistical and Analytical Methods 30

9.2.1 Analysis Plan 30

9.2.1.1 Definition of Analysis Populations 31

9.2.1.2 Exclusion of Data from Analysis 31

9.2.1.3 Safety Data Analysis 31

9.2.1.4 Interim Analysis 32

9.3 Sample Size 32

10. Data Quality Assurance 32

11. Publication 32

12. Study Committees 32

references 33

Part II: ethics and General Study Administration 46

13. Ethical aspects 47

13.1 Local Regulations/Declaration of Helsinki 47

13.2 Informed Consent 47

13.3 Independent Ethics Committees/Institutional Review Board 47

14. conditions for modifying the protocol 48

15. Conditions for terminating the study 48

16. Study documentation, CRFs and record keeping 48

16.1 Investigator's Files / Retention of Documents 48

16.2 Source Documents and Background Data 49

16.3 Inspections 49

16.4 Case Report Forms 49

17. monitoring the study 49

18. confidentiality of trial documents and subjecT records 49

19. publication of data and Protection of trade secrets [50](#__RefHeading___Toc138151618)

LIST OF APPENDICES Page

Appendix 1 Child-Pugh Classification of Severity of Liver Disease [35](#__RefHeading___Toc122877008)

Appendix 2 Pegasys Dose Adjustment Guidelines [35](#__RefHeading___Toc122877008)

Appendix 3 Copegus Intended Dose and Dose Adjustment Guidelines 38

Appendix 4 Intervention Guidelines in the Event of Anemia 41

Appendix 5 Instructions for Sample Processing and Shipment for RibaC 42

| Glossary Of Abbreviations | |
| --- | --- |
| Ab | Antibody |
| AE | Adverse event |
| AFP | Alpha-1-fetoprotein |
| Ag | Antigen |
| ALT (SGPT) | Alanine aminotransferase |
| α1 AT | Alpha 1 antitrypsin |
| AMA | Anti-mitochondrial antibodies |
| ANA | Anti-nuclear antibodies |
| AP | Alkaline phosphatase |
| AST (SGOT) | Aspartate aminotransferase |
| ASMA | Anti-smooth muscle antibodies |
| b.i.d. | Twice daily |
| BP | Blood pressure |
| CHC | Chronic hepatitis C |
| CI | Confidence interval |
| CMV | Cytomegalovirus |
| CRF | Case Report Form(s) |
| DNA | Deoxyribonucleic acid |
| ECG | Electrocardiogram |
| EOT | End of Treatment |
| HAV | Hepatitis A virus |
| HBc | Hepatitis B core |
| HbsAg | Hepatitis B surface antigen |
| HCV | Hepatitis C virus |
| HIV | Human immunodeficiency virus |
| ICH | International Conference on Harmonization |
| IDB | Investigational Drug Brochure |
| IEC | Independent Ethics Committee |
| Glossary Of Abbreviations (cont’d) | |
| IFN | Interferon alpha |
| IRB | Institutional Review Board |
| IgM | Immunoglobulin M antibody |
| IPT | Interferon gamma inducible protein |
| ITT | Intent to treat |
| MIU | Million International Units |
| MRI | Magnetic Resonance Imaging |
| mRNA | Messenger ribonucleic acid |
| OAS | 2’5’-Oligoadenylate synthetase |
| PCR | Polymerase chain reaction |
| PEGASYS® | Pegylated-Interferon-alpha-2a 40 KD |
| RBV | Ribavirin |
| RNA | Ribonucleic acid |
| RBC | Red blood count |
| SAE | Serious adverse event |
| Sc | Subcutaneous |
| SVR | Sustained Virological Response |
| Tiw | Three times per week |
| T_4_ | Thyroxin |
| TSH | Thyroid-stimulating hormone |
| ULN | Upper limit of normal |
| WBC | White blood count |
| VRVR | Very Rapid Virologic Response |
| RVR | Rapid Virologic Respose |
| cEVR | Complete Early Virologic Response |
| pEVR | Partial Early Virologic Response |

# Part I: Study design and conduct

# background and rationale

## Background

Hepatitis C virus (HCV) accounts for approximately 70% of cases of chronic hepatitis in industrialized countries. If left untreated patients may develop cirrhosis, end-stage liver disease, portal hypertension and hepatocellular carcinoma (1, 2).

The response to therapy is greatly related to HCV genotype. Seven genotypes of HCV have been currently been identified. Genotype 1 (G1) is the most common in the Nordic countries, and occurs in approximately 40-50% of cases. Genotypes 2 and 3 (G2/3) contribute an additional 40-50%, and genotypes 4-7 constitute the remaining 5%.

## Study Medications

The current recommended treatment for patients with chronic hepatitis C (CHC) is the combination of Pegylated interferon-alfa (PEG-IFN) and ribavirin (RBV) (3). Interferon-alfa was the first drug shown to have bioactivity against HCV. Combining RBV with interferon-alpha was found to be more effective than interferon-alpha monotherapy in previously untreated patients as well in patients who relapsed after one or more courses of interferon-alpha therapy (4).

### PEGASYS® (Peginterferon-alpha-2a, PEG-IFN 2a)

The interferon-alpha-2a molecule has been chemically modified by Hoffmann-La Roche with a covalent attachment of a branched methoxy polyethylene glycol moiety (5). PEGASYS® (Peginterferon-alpha-2a, PEG-IFN alfa 2a) has a decreased systemic clearance rate and an approximately 10-fold increase in serum half-life compared with interferon-alpha-2a, so that PEGASYS circulates in the blood much longer than does the parent compound. The biological activity of PEGASYS, as measured using serum 2’5’-oligoadenylate synthetase (OAS) activity, is similarly prolonged, resulting in a significantly improved pharmacodynamic profile compared with that of interferon. These observations and additional pharmacokinetic and pharmacodynamic data obtained from animals and healthy male volunteers suggested that PEGASYS injected once per week might be more efficacious than interferon injected three times per week (tiw) (6). Indeed, this hypothesis was confirmed in three large clinical trials in over 1400 patients where treatment with PEGASYS 180 µg once weekly was shown to be more efficacious than treatment with interferon-alpha-2a thrice weekly (5).

### Copegus® (Ribavirin)

Copegus® (RBV) is a guanosine analogue that inhibits the in vivo replication of a wide range of RNA and DNA viruses (7). The mechanism of antiviral activity is not fully defined, although it may involve alteration of cellular nucleotide pools and inhibition of viral RNA synthesis (8). RBV monotherapy has little effect on the replication of HCV but can result in normalization of serum ALT activity and improvement in liver histology. However, relapse occurs in nearly all patients treated with RBV alone (9, 10).

Combining Copegus with PEGASYS has been found to be more effective than PEGASYS monotherapy in the treatment of CHC (5). In a large clinical trial of 1121 patients, a sustained virological response (SVR) was achieved in 53% of patients treated with PEGASYS plus Copegus as compared to 29% of patients treated with PEGASYS alone.

## Clinical Experience

### Combination therapy of Peginterferon-alpha-2a and Ribavirin

The safety and efficacy of PEGASYS in combination with COPEGUS for treatment of hepatitis C infection were assessed in two randomized controlled clinical trials (11, 12). Based on the results of these trials, PEGASYS in combination with COPEGUS is indicated for treatment of adults with chronic hepatitis C infection who have compensated liver disease. It has long been recognized that patients with hepatitis C virus (HCV) genotype 2 or 3 respond better to interferon (IFN) treatment than patients infected with HCV genotype 1, and that patients achieving higher ribavirin concentrations are more likely to achieve SVR (13). Additionally it has recently been reported that higher ribavirin concentrations already on the first day of therapy is associated with improved outcome (14).

## Rationale

### Rationale for the Study Design

Recently it has been reported that higher ribavirin concentrations as early as the first day of combination therapy for HCV as well as higher that standard dosing of ribavirin is associated with improved outcome (18). Additionally because of the long half-life of ribavirin, it takes greater than 4 weeks to achieve steady-state concentrations. In this pilot study, we will evaluate two potentially new strategies to achieve higher initial ribavirin concentrations. The first strategy entails giving a higher initial dose of ribavirin during the first two weeks (“loading”) similar to current clinical practice for many antibiotics. Moreover, a ribavirin concentration after 4 weeks of therapy will be available to the treating physician, enabling possible dose adjustments in the event of suboptimal concentrations. The second strategy encompasses giving mono-therapy with ribavirin for 4 weeks prior to initiating therapy with pegylated interferon (“priming”). In this treatment arm a ribavirin concentration after 4 weeks will also be available as in Group A above. Both of these strategies will be compared to a control group receiving standard-of-care dosing of ribavirin, where no ribavirin concentration is available during therapy as is often the case in current clinical practice.

### Viral kinetics

A large combination trial including 1121 patients, with 453 patients receiving PEGASYS 180 µg/ week and 1000-1200 mg Copegus, with a treatment duration of 48 weeks was the first study to show that a virological response at week 12, defined as a 2-log decrease from base-line HCV-RNA levels or no detectable serum HCV RNA, is a strong predictor of a SVR (11). In this study 86 % of the patients had an early virological response and subsequently 65 % of these patients went on to have a SVR response. Ninety-seven percent (97 %) of patients with HCV genotype 2/3 had an early virological response and 77 % of these patients became a SVR.

There have also been studies looking into rapid virological response at week 4 as a predictor of sustained virological response. In a large combination trial including 1284 patients with chronic hepatitis C, patients received PEGASYS 180 µg/ week in combination with 800 or 1000-1200 mg Copegus per day for 24 or 48 weeks (12). 94% of the patients with HCV genotype 2/3 had a virological response by week 4, and 87 % of these patients had a SVR as compared to 17% SVR for the patients without a virological response by week 4.

In this study, HCV-RNA levels in plasma during the first 12 weeks of therapy will be utilized to evaluate the efficacy of therapy in the 3 treatment arms.

### Liver Fibrosis and Inflammation Score

Liver biopsy is currently considered the gold standard for assessing liver necroinflammation and fibrosis in patients with CHC. It is though a costly an invasive procedure with a risk of complications (15) and the risk of sampling error (16). In a recent retrospective, single-center study by Islam *et al.* (17) a novel index, normalized aspartate amininotransferase (AST), PK-INR, and platelet count (Gothenburg University Cirrhosis Index; GUCI), in predicting liver fibrosis and cirrhosis was introduced. The index uses objective routine laboratory data and is calculated using a simple formula. GUCI is reported to accurately exclude the likihood of cirrhosis if this index is below 1.0. Since liver biopsies will be encouraged but not mandatory in this trial, GUCI will be used as a marker of the fibrosis stage to stratify patients. If a liver biopsy within 2 years of initiation of therapy in this trial has been obtained, central staging of fibrosis and grading of inflammation and steatosis will be performed.

### Rational for Dosage Selection

#### Pegasys

The dose chosen for PEGASYS® (180 μg, sc, once per week) is the dose currently approved in EU for combination therapy with COPEGUS (5).

#### Ribavirin

The dose chosen for COPEGUS 13 mg/kg body weight/day is based on the current Swedish National Treatment Guidelines, and is the current standard of care in Sweden for HCV genotype 1 infected patients (18). Patients in Group A (“Loading”) will receive a double dose the first 2 weeks of combination therapy followed by standard 13 mg/kg body weight/day. And patients in Group B (“Priming”) will receive standard 13 mg/kg body weight/day dosing of ribavirin for four weeks prior to initiating pegylated interferon, which will be maintained during the ensuing 48 week treatment period. Ribavirin should be administered twice-daily with food.

# OBJECTIVES OF The STUDY

## Primary Objective

To evaluate the efficacy of (A) 2 weeks of high dose of ribavirin (“loading”, ≥26 mg/kg/day for 14 days followed by ≥13 mg/kg/day) vs. (B) 4 weeks of ribavirin dosing before initiation of PEG-interferon dosing (“priming”, ≥13 mg/kg/day) followed by concentration targeted (≥ 2.5 mg/L (10.25 μmol/L) 28 days after initiation of ribavirin therapy) dosing of ribavirin in combination with peginterferon alpha-2a in interferon naïve patients with chronic hepatitis C (CHC) virus genotype 1 infection as compared to (C) standard-of-care dosing of ribavirin (≥13 mg/kg/day without monitoring of ribavirin concentrations) in combination with peginterferon alpha-2a as evaluated by the early viral kinetic response and SVR measured by effect on the initial decline of HCV-RNA (during the first days after initiating peginterferon and ribavirin therapy) and second phase decline (day 7 to week 12 of therapy).

## Secondary Objectives

To prospectively evaluate:

- the efficacy of 2 week loading or 4 week priming followed by concentration targeted dosing of ribavirin in combination with peginterferon alpha-2a in interferon naïve patients with chronic hepatitis C (CHC) virus genotype 1 infection as compared to standard-of-care dosing of ribavirin in combination with peginterferon alpha-2a as evaluated by the proportion of patients achieving VRVR ( “very rapid virologic response” i.e. HCV-RNA below 1000 U/mL at treatment day 7 after intiation of PEG-interferon), RVR (“rapid virologic response”, undetectable HCV-RNA at treatment day 28 after intiation of PEG-interferon), cEVR (“complete early virologic response”, i.e. undetectable HCV-RNA at treatment week 12 after intiation of PEG-interferon), and pEVR (“partial early virologic response”, i.e. decline of HCV-RNA by at least 2 log10 compared with baseline at treatment week 12 after intiation of PEG-interferon).
- the efficacy of 2 week loading or 4 week priming followed by concentration targeted dosing of ribavirin in combination with peginterferon alpha-2a in interferon naïve patients with chronic hepatitis C (CHC) virus genotype 1 infection is superior to standard-of-care dosing of ribavirin, peginterferon alpha-2a therapy as evaluated by sustained virological response (SVR, undetectable HCV-RNA 24 weeks after end of treatment).
- the predictive value of monitoring of viral load at day 0 (before the 1^st^ dose of peginterferon alpha-2a), day 3, day 7 (before the 2^nd^ dose of peginterferon alpha-2a), and day 28 (before the 5^th^ dose of peginterferon alpha-2a) for determining which patients will obtain a sustained virological response (SVR) when treated with ribavirin and peginterferon alpha-2a therapy.
- the association between the trough concentrations of ribavirin (day 1, day 3, day 7, day 28, week 8, week 12, week 16, and end-of-treatment), and the therapeutic efficacy of ribavirin and peginterferon alpha-2a therapy.
- The association between plasma IP-10 (days 0, 1, 3, 7, and 14, and weeks 8, 18, and 24), and the therapeutic efficacy of ribavirin and peginterferon alpha-2a therapy.
- the effect of IL-28B polymorfism on the viral kinetic response and SVR.
- the effect of baseline vitamin D concentrations on the viral kinetic response and SVR.
- the association between liver histology as evaluated by the Ishak scoring system (modified HAI score) and steatosis grading, and the therapeutic efficacy of peginterferon alfa-2 combination therapy with ribavirin.
- the association between liver stiffness as evaluated by the FibroScan and the therapeutic efficacy of peginterferon alfa-2 combination therapy with ribavirin.
- the association between liver fibrosis as evaluated by the GUCI and APRI indexes as well as hyaluronic acid, and the therapeutic efficacy of peginterferon alfa-2 combination therapy with ribavirin.
- the association between body mass index (BMI), waist circumference, weight and age, and the therapeutic efficacy of peginterferon alfa-2 combination therapy with ribavirin.
- the predictive value of monitoring CD56 negative NK, CD4^+^ CD38^+^, CD8^+^ CD38^+^, CD8^+^CD16^+^, Foxp3^+^, and pDC (CD303^+^) cells in peripheral blood and liver biopsies as analyzed by FACS prior to and after the initiation of therapy for determining which patients will obtain a sustained virological response (SVR) when treated with peginterferon alfa-2 and ribavirin combination therapy.
- the predictive value of quantifying mRNA expression in pre-treatment liver biopsies for IFI-27, IP-10, CXXL-6, IL-2, IL-8, IL-10, γ-IFN, TNF-α, KRT19, COL1A1, MMP7, TIMP1, FASL, AIF1 for determining which patients will obtain a sustained virological response (SVR) when treated with peginterferon alfa-2 and ribavirin combination therapy.

Others:

To prospectively evaluate:

- the predictive value of monitoring the trough concentrations of ribavirin (day 1, day 3, day 7), and the final steady-state concentration of ribavirin.
- the safety of peginterferon alpha-2a combination therapy with concentration targeted dosing of ribavirin in combination peginterferon alpha-2a, based on accumulated number of adverse events and severe adverse events as well as the impact on quality of life during treatment and follow-up phase.
- the safety of peginterferon alpha-2a combination therapy with concentration targeted dosing of ribavirin in combination peginterferon alpha-2a, with particular regards to the number of patients in each treatment arm developing anemia grade 1 (10.5-9.5 g/dL), grade 2 (9.4-8.0 g/dL), grade 3 (7.9-6.5 g/dL), and grade 4 (<6.5 g/dL).

# STUDY design

## Overview of Study Design and Dosing Regimen

This study will be conducted as a phase III, open label, multicenter trial. A total of 105 patients will be randomized to one of three possible treatment groups.

**Group A:** (“Loading”): PEG-IFN α-2a 180 μg/week plus loading (≥26 mg/kg/day for 2 weeks followed by ≥13 mg/kg/day) and concentration targeted (≥ 2.5 mg/L (10.25 μmol/L) 28 days after initiation of ribavirin therapy) dosing of ribavirin and response guided treatment duration (RVR 24 weeks, non-RVR 48 weeks, pEVR consider 72 weeks), follow-up period 24 weeks.

**Group B**: (“Priming”): Standard-of-care dosing of ribavirin (≥13 mg/kg/day) without PEG-IFN for 4 weeks followed by 24-48 additional weeks of PEG-IFN α-2a 180 μg/week plus standard-of-care dosing of ribavirin (≥13 mg/kg/day) and concentration targeted (≥ 2.5 mg/L (10.25 μmol/L) 28 days after initiation of ribavirin therapy) dosing of ribavirin and response guided treatment duration (RVR 28 weeks, non-RVR 52 weeks, pEVR consider 76 weeks), follow-up period 24 weeks.

**Group C:** (“Standard-of-Care”): PEG-IFN α-2a 180 μg/week plus standard-of-care dosing of ribavirin (≥13 mg/kg/day without any measurement of ribavirin concentration) and response guided treatment duration (RVR 24 weeks, non-RVR 48 weeks, pEVR consider 72 weeks), follow-up period 24 weeks.

## Number of Patients

A total of 105 patients will be enrolled in the study: 35 patients in group A, 35 patients in group B and 35 patients in group C.

## Centers

This study will be conducted at experienced centers in Sweden, Norway, Denmark, and Finland willing to include at least 5 patients. Only those centers qualified by training and experience in the clinical and research management of patients with CHC will be invited to participate.

# STUDY POPULATION

## Target Population

Men and women ≥ 18 years old with CHC genotype 1 will be enrolled in this study. Patients must have HCV-RNA ≥ 15 IU/mL, and compensated liver disease (Child-Pugh Grade A). Patients with other forms of liver disease, human immunodeficiency virus (HIV) infection, hepatocellular carcinoma, anemia, pre-existing severe depression or other psychiatric disease, significant cardiac disease, renal disease, seizure disorders, or severe retinopathy will not be included.

## Inclusion Criteria

To be eligible for this trial, patients must have the following documented:

Inclusion Criteria:

- Written informed consent
- Male and female patients ≥18 years of age
- Serologic evidence of chronic hepatitis C infection by an anti-HCV antibody test
- Serum HCV-RNA ≥15 IU/mL.
- HCV genotype 1 infection confirmed within the past 2 years preceding the initiation of test drug dosing.
- Compensated liver disease (Child-Pugh Grade A clinical classification)
- Patients with cirrhosis or transition to cirrhosis must have an abdominal ultrasound, CT scan, or MRI scan without evidence of hepatocellular carcinoma and a serum AFP ≤100 ng/mL within 2 months of randomization
- Negative urine or blood pregnancy test (for women of childbearing potential) documented within the 24-hour period prior to the first dose of study drug
- All fertile males and females receiving ribavirin must be using effective contraception during treatment and during 4 months for female patients / 7 months for male patients after end of treatment
- Subject must weigh between 45 and 105 kg at screening

## Exclusion Criteria

Patients with any of the following will not be eligible for participation:

Exclusion Criteria:

- Women with ongoing pregnancy or breast feeding
- IFN/ peg-interferon with or without ribavirin therapy at any previous time
- Therapy with any systemic anti-viral, anti-neoplastic or immunomodulatory treatment (including supraphysiologic doses of steroids and radiation) ≤6 months prior to the first dose of study drug
- Any investigational drug ≤6 weeks prior to the first dose of study drug.
- HCV genotype 2, 3, 4, 5, 6, or 7 infection.
- Positive test at screening for anti-HAV IgM Ab, HBsAg, anti-HBc IgM Ab, anti-HIV Ab
- Evidence of a medical condition associated with chronic liver disease other than HCV (e.g., hemochromatosis, autoimmune hepatitis, metabolic liver disease, alcoholic liver disease, toxin exposures)
- History or other evidence of decompensated liver disease
- Neutrophil count <1500 cells/mm^3^ or platelet count <90,000 cells/mm^3^ at screening
- Serum creatinine level >2 mg/dl (>124 µmol/L) or creatinine clearance ≤50 ml/minute at screening
- Severe psychiatric disease, especially depression, as judged by the treating physician.
- History of a severe seizure disorder or current anticonvulsant use
- History of immunologically mediated disease, severe chronic pulmonary disease associated with functional limitation, severe cardiac disease, major organ transplantation or other evidence of severe illness, malignancy, or any other conditions which would make the patient, in the opinion of the investigator, unsuitable for the study
- Thyroid dysfunction not adequately controlled (TSH and T4 levels out of normal range)
- Evidence of severe retinopathy (e.g. CMV retinitis, macula degeneration) or clinically relevant ophthalmological disorder due to diabetes mellitus or hypertension
- Evidence of drug abuse (including excessive alcohol consumption) in accordance with local therapeutic traditions.
- Inability or unwillingness to provide informed consent or abide by the requirements of the study
- Male partners of women who are pregnant
- Hemoglobin <12 g/dL in women or <13 g/dL in men at screening.
- Any patient with an increased baseline risk for anemia (e.g. thalassemia major, spherocytosis, history of GI bleeding, etc) or for whom anemia would be medically problematic coagulopathy.
- Patients with documented or presumed coronary artery disease or cerebrovascular disease should not be enrolled if, in the judgment of the investigator, an acute decrease in hemoglobin by up to 4 g/dL (as may be seen with ribavirin therapy) would not be well-tolerated
- Evidence of allergy to PEG-IFN or ribavirin.

## Concomitant Medication and Treatment

Systemic antiviral, anti-neoplastic and immunomodulatory treatments (including steroids and radiation) are not allowed during the study. Other investigational drugs against HCV are excluded.

Erythropoetin and/or filrgrastim are allowed as concomitant medication to manage side effects during treatment.

The total daily dose of acetaminophen (paracetamol) should not exceed 4 grams per day.

For patients receiving both theophylline and PEGASYS®,,Theofylline levels should be monitored and appropriate dose adjustment should be considered.

A complete listing of all concomitant drugs received must be recorded in the CRF.

**Paracetamol medication less than or equal to 4 gram/day should not be recorded in the CRF.**

**Ibuprofen medication less than or equal to 1.2 g/day should not be recorded in the CRF**

Alcohol consumption is to be strongly discouraged during the study. This is because of possible adverse effects of alcohol on the response to therapy with IFNs in CHC. Patients will be queried on a regular basis concerning their alcohol consumption.

# SCHEDULE OF ASSESSMENTS AND PROCEDURES

## Schedule of Assessments

Baseline assessments should be obtained on the first day of drug administration prior to the initiation of antiviral therapy with PEGASYS and Copegus. This study will employ a serum- and plasma bank for the event that some tests need to be repeated and for herein described analysis.

## Screening Examination and Eligibility Screening Form

The following screening assessments (Table ) must be obtained within 56 days prior to the initiation of test drug administration.

Table 4 Screening Assessments

| Medical History Vital Signs And Physical Examination | Height, waist measurement, vital signs (systolic and diastolic blood pressure, heart rate, and body weight) and ophthalmological moscopic examination according to local standard |
| --- | --- |
| Clinical Chemistry | ALT, AST, gamma GT, total bilirubin, alkaline phosphatase, Calcium, albumin, creatinine, uric acid, cholesterol*, triglycerides*, glucose*, HbA1c*, Ferritin, Iron, TIBC, vitamin B-12, Folic Acid,  Ceruloplasmin†, alpha_1_-antitrypsin† |
| Hematology | Hemoglobin, hematocrit, erythrocyte volume (ery-MCV), reticulocytes, leukocytes, neutrophils, basophils, eosinophils, monocytes, lymphocytes, platelets, prothrombin time International Normalized Ratio (PK-INR) |
| Virology | HCV genotype, quantitative HCV-RNA (**COBAS TaqMan)** |
| Immunology | Anti-HAV IgM Ab, anti-HBc IgM Ab, HBsAg, anti-HCV Ab, anti-HIV Ab, anti-mitochondrial Abs, anti-nuclear Abs, anti-smooth muscle Abs |
| Thyroid Function Tests | TSH^+^, T_4_ (free) |
| Urinanalysis | Dipstick with subsequent microscopic evaluation if positive for hemoglobin |
| HCG Pregnancy Test | For women of childbearing potential, a negative urine (or serum) HCG test needs to be documented **within 24 hours prior** to the first dose |
| Liver Biopsy (optional) | Within 2 years prior to this study is encouraged, but optional in accordance with local treatment traditions |
| Liver ultrasound or CT or MRI | For patients with cirrhosis or transition to cirrhosis |
| Alpha-1-fetoprotein  (optional) | For patients with cirrhosis or transition to cirrhosis |

^+^For patients who exhibit elevated TSH the investigator should consider obtaining an anti-thyroid peroxidase antibody titre.

* Fasting values for cholesterol, triglycerides, glucose and HbA1c

The following examinations should be considered for certain patients at risk in order to establish compliance with the inclusion/exclusion criteria:

| Electrocardiogram | Only for men ≥40 years of age, women ≥50 years of age, and anyone else with a history of pre-existing cardiac disease |
| --- | --- |
| Immunology | Ceruloplasmin†, alpha_1_-antitrypsin† |

Notes:

†Ceruloplasmin and alpha_1_-antitrypsin do not need to be evaluated if these have ever been documented to be normal in the past

Patients at risk of ophthalmologic disorders (e.g diabetes mellitus and hypertension), with mild to moderate retinopathy should at screening have a thorough eye examination, including fundoscopic examination, should be performed to establish a baseline prior to treatment in this study.

Patients with history of psychiatric disease

Pegylated interferon should be used with caution in patients who report a history of depression. Physicians should monitor all patients for evidence of depression and should inform patients of the possible development of depression prior to initiation of pegylated interferon therapy. Patients should report any sign or symptom of depression immediately.

## Study Assessments

### Efficacy Assessments

Efficacy assessments consist of serum HCV-RNA via PCR and ALT determination.

Quantitative viral titers (COBAS TaqMan 48™)) will be obtained during screening, day 28, week 12, week 24, End-of-Treatment, and 24 weeks post-end of treatment and sent to local lab for assessment.

Patients who discontinue from treatment prematurely, are expected to return for sampling for HCV RNA at end-of-treatment, and 24 weeks post-end of treatment.

###### Viral Kinetic

Quantitative viral titers (COBAS TaqMan 48™) will be obtained at screening, day 0, day 3, day 7, day 28, week 12, End-of-Treatment, and 24 weeks post-end of treatment and sent to central lab for analysis. Group B (“Priming”) will also obtain a plasma sample on day -28 (before the first “priming” dose of ribavirin) for analysis at the central lab.

Instructions for sampling and shipment can be found in Appendix.

**Ribavirin Plasma Concentration**

Trough plasma concentrations of ribavirin will be obtained before the morning dose of ribavirin day 1, day 3, day 7, day, 14, day 28, week 8, week 12, week 18, End-of-Treatment. Group B (“Priming”) will also obtain trough plasma sample for ribavirin day -27 (the first day after initiation of “priming” dose of ribavirin) and day -21 (one week after initiation of “priming” dose of ribavirin).

Instructions for sampling and shipment can be found in Appendix.

**IP-10**

During screening and on days 0, 1, 3, 7, and 14, and weeks 8, 18, and 24 plasma will be drawn for later evaluation of IP-10 concentrations.

**IL-28B Polymorfism**

During the screening visit an EDTA blod sample will be drawn for later evaluation of IL-28B polymorfism and the impact on outcome.

**Vitamin D**

During the screening period a serum sample will be drawn for later analysis of Vitamin D (25 hydroxy-vitamin D) at a central laboratory.

###### Liver biopsy (if obtained)

Four or more unstained slides of and/or paraffin embedded liver biopsies (archived or taken during screening) are together with the local pathologist evaluation sent to Sahlgrenska University Hospital, Gothenburg, Sweden for assessment.

If a liver biopsy is obtained during the screening period, it is encouraged that 1 cm liver tissue be placed in Allprotect Tissue Reagent (Qiagen cat. No. 76405) and immediately frozen at -70°C for later evaluation of the predictive value of quantifying mRNA expression for IFI-27, IP-10, CXXL-6, IL-2, IL-8, IL-10, γ-IFN, TNF-α, KRT19, COL1A1, MMP7, TIMP1, FASL, AIF1 for determining which patients will obtain a sustained virological response (SVR) when treated with peginterferon alfa-2 and ribavirin combination therapy.

Instructions for sampling and shipment can be found in Appendix

**FibroScan (optional)**

FibroSacn evaluations are optional but encouraged during the screening period as well as week 24 after completion of therapy.

**PBMCs during screening (optional)**

If possible sampling for PBMC’s during screening is encouraged in order to evaluate the predictive value of monitoring CD56 negative NK, CD4^+^ CD38^+^, CD8^+^ CD38^+^, CD8^+^CD16^+^, Foxp3^+^, and pDC (CD303^+^) cells in peripheral blood and liver biopsies as analyzed by FACS prior to and after the initiation of therapy for determining which patients will obtain a sustained virological response (SVR) when treated with peginterferon alfa-2 and ribavirin combination therapy.

Instructions for sampling and shipment can be found in Appendix

### Safety Assessments

Safety assessments will be performed throughout the treatment period and the follow-up period as outlined in the Schedule of Assessments (Section 5.1). Measures of safety will consist of:

- Vital signs consisting of systolic and diastolic blood pressure, heart rate, and body weight
- Clinical chemistry including AST, gamma-GT, total bilirubin, alkaline phosphatase, albumin, creatinine, and uric acid
- Serum ALT activities at each visit
- Hematology including complete (hemoglobin, reticulocytes, WBC, platelets) and differential blood count
- Urinalysis consisting of protein, blood, and glucose examinations
- Thyroid function test
- Women of child-bearing potential need to have a urine or serum **pregnancy test** (a) within 24 hours prior to first dose (all female patients) (b) every study visit while on test drug therapy and (c) for 24 weeks following the last dose of test medication. Investigators must ensure that any patient who is discontinued from study medication prematurely has the follow-up pregnancy tests performed for the full 24 weeks following the last dose of study medication.
- Ophthalmologic examination: Patients with preexisting ophthalmologic disorders (e.g. diabetic or hypertensive retinopathy) should receive periodic ophthalmologic exams during therapy. Pegylated interferon treatment should be discontinued in patients who develop new or worsening ophthalmologic disorders. Any patient complaining of decrease or loss of vision must have an eye examination
- Documentation of concomitant medication
- Documentation of dose adjustments and premature withdrawals for safety reasons or intolerance.
- Documentation of clinical adverse events (AEs).
- An independent DSMB will evaluate safety after the first 30 patients have reached treatment weeks 2 and 12.
- The use of erythropoietin (NeoRecormon) ≥3000 IE 3 times week sc is permitted throughout the study at the discretion of the treating physician with the exception if the patient has any of the following exclusion criteria for the use of erythropoietin: platelet count >500,000, risk of thrombosis, or poorly controlled hypertension, especially if hemoglobin decreases persistently to < 10.0 g/dL or in the event of a rapid decrease in hemoglobin exceeding 4.0 g/dL over a 2 week period. If erythropoietin is initiated, a new ribavirin concentration at local lab should be evaluated after 4 weeks in Groups A (“Loading”) and B (“Priming”).
- In the event of anemia, dose reductions of ribavirin are permitted in all study arms throughout the study at the discretion of the treating physician.
- If hemoglobin decreases to < 8.0 g/dL, ribavirin should be discontinued.
- If hemoglobin decreases to < 7.0 g/dL, blood transfusion should be considered at the discretion of the treating physician.
- If hemoglobin decreases to < 6.5 g/dL, blood transfusion should be given.
- Patients will discontinue therapy if the decrease in plasma HCV RNA between week 12 and baseline is ≤ 2 log_10_ IU/mL or if plasma HCV RNA is still detectable by treatment week 24.
- The members of the Steering Committee in each respective country will be responsible for monitoring the participating centers in their country. One or more experienced monitor is recommended per participating country.
- Only experienced treatment centers willing to include ≥ 5 patients will be permitted to participate in the study.
- All serious adverse events in the study will be reported to IST and Welwyn.

Patients will also be assessed for safety after their last dose of test medication following premature discontinuation from the study. This includes a laboratory evaluation at least 12 weeks following the final dose of test drug (for details please refer to section 3.1). Generally patients should be encouraged to stay in the trial. Counseling about contraception and behaviors associated with an increased risk of pregnancy must be repeated on a monthly basis.

# end points of the study

## Primary Endpoint

The early virological response as measures by the decline in HCV-RNA during the first 12 weeks (in particular the first and second phase decline) of peginterferon alpha-2a and ribavirin therapy in the three study arms.

## Secondary Efficacy Endpoints

## Percentage of patients with non-detectable HCV-RNA at end the end of 24 week or 48 week of treatment as measured by Roche COBAS TaqMan

## VRVR, RVR, cEVR, and pEVR rates defined as percentage of patients achieving these goals as measured by TaqMan PCR in the three study arms.

## Percentage of patients with non-detectable HCV-RNA at study day 1, 3, 7, 14, 21, 28, week 8, week 12, end of treatment, and 24 weeks after completion of treatment as measured by TaqMan PCR as compared to day 0.

## SVR rate defined as percentage of patients with non-detectable HCV-RNA as measured by TaqMan PCR at 24 weeks post completion of the treatment period in the three study arms.

## Relapse rates defined as percentage of patients with non-detectable HCV-RNA as measured by TaqMan PCR at the end-of-treatment but with detectable HCV-RNA 24 weeks post completion of the treatment period in the three study arms.

## SVR rate and percentage of patients with normal serum ALT levels at 24 weeks after completion of the treatment period in the total patient population, and its association with the following factors:

## early virological response

## pharmacokinetic response to ribavirin

# STUDY MEDICATIONS

## Dose and Schedule of Study Medications

Patients will receive PEGASYS® 180 μg in 0.5 mL (prefilled syringes) administered sc once weekly. Specific guidelines for adjusting the dose of PEGASYS® are provided in Section 8.3. All PEGASYS® administrations will be via the sc route utilizing sterile technique. Test drug may be self-administered by the patients, except for the first dose, which should be administered in the clinic under the supervision of study personnel. Before providing the patient with test medication, the investigator or a qualified staff member will instruct the patient on the proper methods of storage of the medication, self-injection, and management and disposal of needles and syringes.

Patients will in combination with PEGASYS receive COPEGUS (ribavirin) which is administered p.o. daily: 13 mg/kg body weight. In Group A, patients will receive COPEGUS (ribavirin) 26 mg/kg body weight for the first 2 weeks (“Loading”) followed by COPEGUS (ribavirin) 13 mg/kg body weight for the remaining treatment duration. In Group B, patients will receive monotherapy with COPEGUS (ribavirin) 13 mg/kg body weight for 4 weeks prior to initiation of PEGASYS combination therapy. In both Group A and B, a ribavirin concentration will be obtained at local lab on day 28 after the initiation of PEGASYS, allowing for dose adjustments as deemed fit by the treating physician with the goal of achieving a ribavirin concentration (≥ 2.5 mg/L or 10.25 μmol/L). In Group C all patients will receive COPEGUS (ribavirin) 13 mg/kg body weight in combination with PEGASYS for the entire treatment duration without a ribavirin concentration without assessment on day 28 (“Standard-of-Care”)

All patients in this study will receive ribavirin treatment with food. By definition, ribavirin, with food entails taking their doses within 1 hour before or 2 hours after a meal. The meal should be considered “regular” as opposed to “fat-restricted”. Dose adjustment guidelines for ribavirin are provided in section 8.3.

## Preparation and Administration of Study Medication

PEGASYS will be supplied to patients in commercial packaging each containing four pre-filled syringes containing 180 µg/ml PEGASYS. Patients should be informed to keep syringes stored refrigerated at 2°-8° Celsius out of reach of children.

Copegus will be supplied to patients in commercial packaging each containing 168 tablets of 200 mg Copegus or 56 tablets of 400 mg . Patients should be informed to keep tablets stored at room temperature (15°-30° Celsius) out of reach of children. Ribavirin should be administered twice-daily with food.

## Blinding and Randomization

This is an open label study with randomization occurring during the screening period, i.e. 4-12 weeks prior to the initiation of pegylated interferon therapy. During randomization patients will be stratified according to gender, age above or below 40 years, and GUCI < or ≥ 1.0 . Randomization will be computerized and will be performed once all inclusion criteria and none of the inclusion criteria have been fulfilled in the e-CRF.

## Compliance

Patients will be asked to record each PEGASYS injection and Copegus dose in a patient diary. Missed or reduced doses should also be recorded. The patient diary should be returned to study site personnel by the end of the study, and be available for inspection at the end of the study.

Missed or reduced doses should be recorded in CRF.

## Treatment Duration

Regardless of treatment arm, all patients with undetectable HCV RNA at week 4 (RVR) as analyzed by the local lab will receive 24 weeks of combination therapy. Similarly patients not achieving RVR will receive 48 weeks of therapy with the exception of patients having detectable HCV RNA at week 12 but not at week 24 (pEVR) where treatment for 72 weeks may be considered if the patient is willing to extend therapy beyond 48 weeks.

## Stopping Rule

Regardless of treatment arm, all patients achieving ≤ 2 log_10_ reduction in HCV RNA by week 12 or having detectable HCV RNA at week 24, will discontinue therapy and be considered as “non-responders” not having achieved SVR.

# SAFETY ISSUES

## Adverse Events and Laboratory Abnormalities

### Clinical Adverse Events

An Adverse Event (AE) is any untoward medical occurrence in a patient or clinical investigation subject administered a pharmaceutical product and which does not necessarily have to have a causal relationship with this treatment. An AE can therefore be any unfavorable and unintended sign (including an abnormal laboratory finding, for example), symptom, or disease temporally associated with the use of a medicinal product, whether or not considered related to the medicinal product. Pre-existing conditions, which worsen during a study are to be reported as AE.

All clinical adverse events (AEs) encountered during the clinical study will be reported on the AE page of the CRF. Intensity of adverse events will be graded on a four‑point scale (mild, moderate, severe, life‑threatening) and reported in detail as indicated on the CRF. Relationship of the adverse event to the treatment should also be assessed.

**Flu-like symptoms**

The following events can be reported as flu-like symptoms: Fatigue, headache, pyrexia, myalgia and rigors

#### Severity

Assessing Adverse Events by Intensity

| **Intensity Level** | **Definition** |
| --- | --- |
| Mild | Discomfort noticed but no disruption of normal daily activity |
| Moderate | Discomfort sufficient to reduce or affect daily activity |
| Severe | Inability to work or perform normal daily activity |
| Life-Threatening | Represents an immediate threat to life |

#### Relationship

Assessing Adverse Events by Relationship to Study Drug Administration

Relationship of an AE to treatment should be assessed using the following terms:

**YES** (Related to test drug)

**NO** (unrelated to test drug)

### Laboratory Test Abnormalities

Laboratory test results will be recorded on the laboratory results pages of the Case Report Form, or appear on electronically produced laboratory reports submitted directly from the central laboratory, if applicable. Laboratory test value abnormalities as such should not be reported on the AE page of the CRF as adverse events unless there is an associated clinical condition for which

a) it is considered to be a Serious Adverse Event (SAE), or

b) the patient is permanently discontinued from study drug because of the abnormal test value, or

c) results in a requirement for additional concomitant treatment or a modification of current concomitant treatment already given for a laboratory abnormality noted at screening/baseline

Neutropenia, thrombocytopenia and anemia should only be reported as an Adverse Events if the following criteria are fulfilled:

**Neutropenia < 500 cells/mm^3^**

**Thrombocytopenia < 25,000 cells/mm^3^**

**Anemia < 8.5 g/dl**

The frequency of laboratory test abnormalities have been thoroughly documented in previous development trials.

## Handling of Safety Parameters

### Serious Adverse Events

Any clinical adverse event or abnormal laboratory test value that is *serious* occurring during the course of the study, irrespectively of the treatment received by the patient, must be reported to the National Health Authority adhering to local requirements. The definition and reporting requirements of the ICH Guideline for Clinical Safety Data Management, Definitions and Standards for Expedited Reporting, Topic E2 will be adhered to.

A **serious** adverse event is any experience that suggests a significant hazard, contraindication, side effect or precaution. With respect to human clinical experience, this includes any experience which

• is fatal or life-threatening;

• requires inpatient hospitalization or prolongation of an existing hospitalization;

• results in persistent or significant disability/ incapacity;

• is a congenital anomaly/ birth defect;

• is medically significant or requires intervention to prevent one or other of the outcomes listed above

Medical and scientific judgment should be exercised in deciding whether expedited reporting is appropriate in other situations, such as important medical events that may not be immediately life-threatening or result in death or hospitalization but may jeopardize the patient or may require intervention to prevent one of the outcomes listed in the definitions above. These situations should also usually be considered serious.

The term **severe** is a measure of **intensity**, thus a severe adverse event is not necessarily **serious**. For example, nausea of several hours’ duration may be rated as severe, but may not be clinically serious.

A **death** occurring during the study or which comes to the attention of the investigator within 4 weeks after stopping the treatment or during the 24 weeks of protocol-defined follow-up period, whether considered treatment-related or not, must be reported.

Any pregnancy occuring during a clinical study with an investigational drug must be reported as an SAE for tracking purposes. All pregnancies identified during this study need to be followed to conclusion and outcome reported.

Female patients should immediately inform the Investigator of any pregnancies and should be instructed by the Investigator to stop taking study medication. (Pregnancies occurring up to 6 months after the completion of the study must also be reported to the Investigator). The Investigator should counsel the patient, discuss the risks of continuing with the pregnancy and the possible effects on the fetus. Monitoring of the patient should continue until the conclusion of the pregnancy.

Pregnancy occurring in the partner of a patient participating in the study should also be reported to the Investigator. (Please refer to section 8.2.4 and 8.5)

Such preliminary reports will be followed by detailed descriptions later, which will include copies of hospital case reports, autopsy reports and other documents when requested and applicable.

For serious and all other AEs, the following must be assessed and recorded on the AE page of the CRF: intensity, relationship to test substance, action taken regarding test substance, and outcome to date.

The investigator must notify the Institutional Review Board (IRB) (Independent Ethics Committee (IEC)) of such an event in writing as soon as is practical and in accordance with international and local laws and regulations.

Note: The definitions for and procedures for reporting SAEs to Health Authorities will be taken from the ICH guidelines.

**All SAEs should be faxed to your local Roche office for notification**

ROCHE LOCAL COUNTRY CONTACT for SAEs

**Please specify:**

### Treatment and Follow-up of Adverse Events

Adverse events, especially those for which the relationship to test drug is “yes”, should be followed up until they have returned to baseline status or stabilized. If the use of medications excluded by the protocol are deemed necessary, the patient may need to be discontinued from the test regimen after consultation with Roche.

### Follow-up of Abnormal Laboratory Test Values

In the event of unexplained abnormal laboratory test values, the tests should be repeated immediately and followed up until they have returned to the normal range and/or an adequate explanation of the abnormality is found. If a clear explanation is established it should be recorded on the CRF.

### Pregnancy

Pregnancy is to be strictly avoided during the course of this trial. However, if a female subject becomes pregnant during the study she must be instructed to stop taking the trial medication and immediately inform the investigator. Pregnancies occurring up to 24 weeks after the completion of the trial medication must also be reported to the investigator. The investigator should counsel the subject, discuss the risks of continuing with the pregnancy and the possible effects on the fetus. Monitoring of the patient should continue until conclusion of the pregnancy.

Pregnancy occurring in the partner of a male patient participating in the study should also be reported to the investigator. The partner should be counseled and followed as above. Such patients may not continue to receive ribavirin.

***Please also refer to section 8.5 “Warnings and Precautions”!***

## Dose Adjustment Guidelines for Intolerance

The intention of the protocol is that patients demonstrating a response to therapy remain on test drug until the completion of the trial. However, it is possible that some patients will encounter transient or prolonged adverse effects at some juncture during their participation in the trial necessitating test drug dosage adjustment. To minimize the effects of these modifications on the eventual evaluation of the safety, tolerability, and efficacy of test drug regimens, the principles in the following sections will be used to adjust the dose of test drugs.

### PEGASYS® Dose Modifications

If at all possible, doses should not be held or eliminated. This recommendation stems from concerns that extended periods of lowered drug concentrations in the blood may be associated with the replication of the more resistant clones of the virus, resulting in a lack of sustained response at the conclusion of therapy.

Specific dose adjustment guidelines for PEGASYS® are provided in appendix 2 for neutropenia, thrombocytopenia and elevated serum ALT activities.

For other adverse effects considered to be possibly related to PEGASYS®, including laboratory abnormalities, adverse events, and vital signs changes, investigators should utilize section 8.3.1.1 below labeled “General Dose Reduction Guidelines”. When practicable, abnormal laboratory results should be confirmed as soon as possible following notification of the investigator. If appropriate, downward adjustments in one level increments should be considered. The lowest dose of PEGASYS® that should be administered is 45 μg. It should be kept in mind that whereas these guidelines should be generally followed to promote consistency across centers, other responses by an investigator may be more appropriate in some circumstances.

Once the patient’s unit dose has been decreased, the investigator may attempt to increase the dose back to or towards that originally assigned if the event or circumstance responsible for the dosage adjustment has resolved or improved.

**If one week or more of PEGASYS**® **dosing is missed (or changed), that information must be recorded on the Case Report Form.**

Decremental adjustments should be uniform across centers and patients. The following downward adjustments should be utilized:

| Assigned Dose | One Level Adjustment | Two Level Adjustment | Three Level Adjustment |
| --- | --- | --- | --- |
| PEGASYS® 180 μg | PEGASYS® 135 μg | PEGASYS® 90 μg | PEGASYS® 45 μg |

#### General Dose Reduction Guidelines

See Appendix 2

### Ribavirin Dose Modifications

If the ribavirin concentration measured on day 28 in Groups A and B is below the target concentration (2.5 mg/L (10.25 μmol/L)), the dose of ribavirin should be increased as soon as possible preferably within 2 weeks of measuring the ribavirin concentration if the patient can tolerate an increase. The new recommended dose is the previously given dose divided by the concentration of ribavirin measured multiplied by the target concentration (2.5 mg/L (10.25 μmol/L)) (see Appendix 3). In the event of a change in ribavirin dosing in Groups A (“Loading”) and B (“Priming”), a new local ribavirin concentration should be evaluated after 4 weeks. Ribavirin should be administered twice-daily with food.

Where dose reduction is required stepwise incremental adjustments of 200 mg (1 tablet) should be utilized where clinical circumstance allows.

If either of the following is confirmed, it is recommended that the ribavirin dose should be reduced, at the discretion of the investigator dependent on local treatment guidelines:

- A patient without significant cardiovascular disease experiences a fall in hemoglobin to <10 g/dL (<100 g/L) and >8.5 g/dL (>85 g/L) or
- A patient with stable cardiovascular disease experiences a fall in hemoglobin by >2 g/dL (>20 g/L) during any 4 weeks of treatment

For the purposes of this protocol, patients with “stable cardiovascular disease” include not only patients with known, stable cardiac and vascular disorders, but also patients with major cardiac risk factors including hypertension and diabetes mellitus.

Patients who have more than a 3 g/dL (30 g/L) decrease from baseline in their Hgb concentration should have an appropriate work-up for anemia, including reticulocyte count, search for sources of bleeding, etc.

Moreover, ribavirin should be discontinued under the following circumstances:

- If a patient without significant cardiovascular disease experiences a fall in hemoglobin confirmed to be less than 8.0 g/dL (80 g/L).
- If a patient with stable cardiovascular disease maintains a hemoglobin value <12 g/dL (<120 g/L) despite 4 weeks on a reduced dose of 600 mg.

Ribavirin monotherapy is not allowed; in the event that PEG-IFN alfa-2a treatment has to be permanently discontinued the patient must cease all study medications and must be followed for safety reasons for at least 12 weeks.

In the event of ribavirin being discontinued, the patient may continue to receive PEG-IFN alfa-2a monotherapy for the rest of the planned treatment period. Ribavirin can be reintroduced, at the investigator´s discretion. Ribavirin may be reintroduced at full dose or in a stepwise manner as indicated in the table above.

The use of erythropoietin (NeoRecormon) ≥3000 IE 3 times week sc is permitted throughout the study at the discretion of the treating physician with the exception if the patient has any of the following exclusion criteria for the use of erythropoietin: platelet count >500,000, risk of thrombosis, or poorly controlled hypertension, especially if hemoglobin decreases persistently to < 10.0 g/dL or in the event of a rapid decrease in hemoglobin exceeding 4.0 g/dL over a 2 week period. If erythropoietin is initiated, a new ribavirin concentration at local lab should be evaluated after 4 weeks in Groups A (“Loading”) and B (“Priming”).

In the event of anemia, dose reductions of ribavirin are permitted in all study arms throughout the study at the discretion of the treating physician.

If hemoglobin decreases to < 8.0 g/dL, ribavirin should be discontinued.

If hemoglobin decreases to < 7.0 g/dL, blood transfusion should be considered at the discretion of the treating physician.

If hemoglobin decreases to < 6.5 g/dL, blood transfusion should be given.

If any dose of ribavirin dosing is missed (or changed), that information must be recorded on the Case Report Form.

## Premature Withdrawal

Patients have the right to withdraw from the study at any time for any reason. The investigator also has the right to withdraw patients from the study if it is in the best interest of the patient, e.g. in the event of intercurrent illness, adverse events, insufficient therapeutic response, protocol violations, administrative reasons or other reasons. An excessive rate of withdrawals can render the study uninterpretable. Therefore, unnecessary withdrawal of subjects should be avoided. Should a subject decide to withdraw, all efforts will be made to complete and report the observations as thoroughly as possible.

The investigator should contact the subject either by telephone or through a personal visit. Alternatively a responsible relative must be contacted to determine as completely as possible the reason for the withdrawal. If the reason for removal of a subject from the study is an adverse event or an abnormal laboratory test result, the principal specific event or test will be recorded on the Case Report Form.

All patients who discontinue from treatment prematurely should be assessed at 12 weeks post their last dose of study medication for safety reasons (see Section 5.3.2).

Patients who discontinue from treatment prematurely after the first 12 treatment weeks and whose HCV RNA is undetectable at last PCR assessment are expected to return for PCR assessments at the end of treatment visit (i.e. as soon as possible after the last dose of study medication) and 24 weeks post end of treatment (see Section 5.3.1).

If necessary, patients may discontinue from ribavirin treatment and remain on PEG-IFN monotherapy. However, because no benefit has been shown from ribavirin monotherapy in the treatment of CHC, patients who discontinue from PEG-IFN must discontinue from the entire treatment regimen (PEG-IFN plus ribavirin).

## Warnings and Precautions

### Peginterferon-alpha-2a

Alpha interferons, including PEG-IFN may cause or aggravate fatal or life-threatening neuropsychiatric, autoimmune, ischemic, and infectious disorders. Patients should be monitored closely with periodic clinical and laboratory evaluations. Therapy should be withdrawn from patients with persistently severe or worsening signs or symptoms of these conditions. In many, but not all cases, these disorders resolve after stopping PEG-IFN therapy.

To date, no teratology or reproduction studies have been conducted in humans with PEG-IFN. Primate teratology studies indicate an increased incidence of spontaneously aborted fetuses in pregnant rhesus monkeys (*Macaca mulatta*) receiving high doses of intramuscular Roferon®-A. Studies with Roferon®-A in non-pregnant rhesus monkeys have shown menstrual cycle irregularities, including prolonged menstrual periods. Male fertility and teratological evaluations have yielded no significant adverse effects up to date. However, fertile men will be excluded from this study unless using effective contraception during the treatment period.

Interferons are human proteins with a substantial degree of species specificity making extrapolation of animal study data to humans of questionable value. Investigations have been conducted in normally cycling healthy women using non-recombinant human leukocyte interferon. Results demonstrate a significant reduction of serum estradiol and progesterone concentrations during the treatment interval.

There are no adequate, controlled studies of any IFN in pregnant women.

Please refer to the Investigator´s Brochure (8) for further information.

### Ribavirin

Ribavirin is a known teratogen (see below for further information). **Therefore, extreme care must be taken to avoid pregnancy during the study in female patients, and female partners of male patients. RIBAVIRIN THERAPY SHOULD NOT BE INITIATED UNLESS A REPORT OF A NEGATIVE PREGNANCY TEST HAS BEEN OBTAINED IMMEDIATELY PRIOR TO INITIATION OF THERAPY.**

Fertile patients will be excluded from this study unless they are using two reliable forms of contraception during the treatment period and follow-up. Females who are pregnant or lactating will also be excluded. A pregnancy test will be performed on each female of childbearing potential prior to entry into the study and must be performed at study visits thereafter during the study. Moreover, fertile females and males should continue to use effective birth control (and fertile females to perform a monthly urine pregnancy test) for 6 months following their last dose of ribavirin.

#### Pregnancy

Ribavirin has a significant teratogenic and/or embryocidal potential in all test animals. These effects were observed even at doses as low as one twentieth of the recommended human dose. Malformations of the skull, palate, eye, jaw, limbs, skeleton, and GI tract were observed; the incidence and severity of teratogenic effects increasing with escalation of the drug dose. Survival of fetuses and offspring was reduced. In conventional embryotoxicity and teratogenicity studies in rats and rabbits, no effect dose levels were well below those for proposed clinical use.

Therefore, in order to avoid pregnancy, females of childbearing age and males should use two reliable forms of effective contraception (combined) throughout the entire period of the study (treatment and follow-up): these may include, but are not limited to, birth control pills, IUDs, condoms, diaphragms, implants, surgical sterilization, or being in a post-menopausal state.

It should also be noted that ribavirin accumulates in spermatozoa (19), i.e. concentrates in the semen. Because ribavirin may be transmitted in semen, it is recommended that one method of contraception be a barrier-type, e.g., condom.

If pregnancy occurs in a patient or partner of a patient during the treatment period, the cases should be handled as described in Section 8.2.4 (“Pregnancy”).

Also, a male patient must immediately report to the investigator any existing or occurring pregnancy in a sexual partner (even if he did not father the child) that occur while he is taking study medication and for 7 months following discontinuation of study medication. Such male patients must not continue to receive ribavirin, which accumulates in spermatozoa. The latter reason also leads to the recommendation of using a contraceptive barrier method in addition to another contraceptive. The partner should be counseled and followed as described above. The male patient will be discontinued from ribavirin (and can continue to receive PEG-IFN) and followed per the provisions of the protocol.

The patient consent form has to include this information with special attention to the potential risk to the fetus should conception occur while the patient or sexual partner is participating in PEG-IFN/IFN and ribavirin trials.

#### Carcinogenesis and Mutagenesis:

Although the literature and sponsor-conducted studies indicate that ribavirin is genotoxic (20), results from the 6-month carcinogenicity study in p53 (+/-) knockout mice revealed no evidence of treatment-related neoplasia. A study in rats to assess the carcinogenic potential of ribavirin is ongoing.

#### Adverse Reaction

The primary toxicity of ribavirin is anemia. Reduction in hemoglobin levels generally occurs within the first 1-2 weeks of initiating therapy. Cardiac and pulmonary events associated with anemia may occur.

Ribavirin should be administered with caution to patients with pre-existing cardiac disease. Patients should be assessed before commencement of therapy and should be appropriately monitored during therapy. If there is any deterioration of cardiovascular status, therapy should be stopped. Ribavirin should be administered with additional precautionary statement for diabetic patients and patients with history of gout to increase the safety of test drug administration

In addition, ribavirin is contraindicated in patients with a history of hypersensitivity to ribavirin.

# STATISTICAL considerations and ANALYtical plan

## Primary and Secondary Study Variables

### Primary Variable

The early virological response as measured by decline in HCV-RNA during the first 12 weeks of peginterferon alpha-2a and ribavirin therapy (especially the 1^st^ and 2^nd^ phase reduction in HCV RNA) in the three study arms.

### Secondary Efficacy Variables

### VRVR, RVR, cEVR, and pEVR rates defined as percentage of patients achieving these goals as measured by TaqMan PCR in the two three arms.

### Percentage of patients with non-detectable HCV-RNA at study day 1, 3, 7, 14, 21, 28, week 8, week 12, end of treatment, and 24 weeks after completion of treatment as measured by TaqMan PCR as compared to day 0.

### SVR rate defined as percentage of patients with non-detectable HCV-RNA as measured by TaqMan PCR at 24 weeks post completion of the treatment period in the three study arms.

### Relapse rates defined as percentage of patients with non-detectable HCV-RNA as measured by TaqMan PCR at the end-of-treatment but with detectable HCV-RNA 24 weeks post completion of the treatment period in the three study arms.

### SVR rate and percentage of patients with normal serum ALT levels at 24 weeks after completion of the treatment period in the total patient population, and its association with the following factors:

### early virological response

### pharmacokinetic response to ribavirin

### Secondary Safety Variables

- Adverse event rate and profile
- Other laboratory tests
- The proportion of patients experiencing anemia grade 1 (10.5-9.5 g/dL), grade 2 (9.4-8.0 g/dL), grade 3 (7.9-6.5 g/dL), and grade 4 (< 6.5 g/dL) will be evaluated in each study arm.

## Statistical and Analytical Methods

### Analysis Plan

This is a pilot trial with the viral kinetic response and SVR measured as described above, during the first 12 weeks of therapy as primary endpoint with the aim of investigating the feasibility and safety of “loading” and “priming” dosing of ribavirin in conjunction with PEG-IFN for chronic HCV genotyp 1. Intention-to-treat and Per-protocol analysis on primary endpoints will be performed for patients receiving at least one dose of the study drugs. Additionally, an analysis of SVR with regards to whether or not the target concentration of ribavirin 2.5 mg/L (10.25 μmol/L) at day 28 was achieved across all study arms will be performed.

#### Definition of Analysis Populations

**Intent-to-treat analysis population** is defined to include all patients who received at least one dose of study medication. Except for change from baseline parameters, the primary and all other secondary efficacy parameters will be analyzed using the intent-to-treat population.

**Per-protocol** **analysis population** is defined to include all patients who received ≥80% of both planned Pegasys and Copegus dose for ≥80% of planned treatment duration.

**Standard analysis (per-protocol) population** will exclude a patient if the patient meets any of the exclusion criteria listed in Section 9.2.2.2. Analysis using the standard population will be performed only if > 5% of patients are excluded from one of the treatment groups.

**Safety analysis population** is defined to include only patients who receive at least one dose of (either) study medication and have at least one post-baseline safety assessment.

#### Exclusion of Data from Analysis

The following patients will be excluded from the standard (per protocol) analysis:

1. Patients who never took any study medication.
2. Patients on PEGASYS® who received ≤80% of both planned PEGASYS®  and COPEGUS® dose for ≤80% of planned treatment duration.
3. Patients with no post-baseline HCV-RNA assessment.

The following patients will be excluded from the safety analysis:

1. Patients who never took any study medication.
2. Patients without any post-baseline safety assessment.

#### Safety Data Analysis

Adverse events will be assigned preferred terms and categorized into body systems according to the Medical Dictionary for Drug Regulatory Affairs (MedDRA) classification of the World Health Organization (WHO) terminology. In the analyses adverse events occurring during treatment and within 168 days of last trial medication will be summarized.

The proportion of patients with reported adverse events during the target period, will be calculated by dividing the number of these patients by the number of patients that can be evaluated for safety. Adverse events will be summarized by study groups mentioned in Section 9.2.2.1, by body system and event within each body system.

Certain potentially clinically relevant laboratory abnormalities will be analyzed in more detail. These laboratory abnormalities include anemia, neutropenia, thrombocytopenia, and elevated ALT levels.

For hemoglobin, neutrophil and platelet counts the lowest value for each patient during treatment and follow-up will be summarized.

#### Interim Analysis

# An independent DSMB will evaluate safety after the first 30 patients have reached treatment weeks 2 and 12.

## Sample Size

This is a pilot trial with the viral kinetic response and SVR measured as described above, during the first 12 weeks of therapy as primary endpoint with the aim of investigating the feasibility and safety of “loading” and “priming” dosing of ribavirin in conjunction with PEG-IFN for chronic HCV genotyp 1. To demonstrate an increase in the reduction of HCV RNA from day 0 to 3 from 0.9 log_10_ IU/mL in Group C (Standard-of-Care) to 1.4 log_10_ IU/mL in Group A or B or an increase in the reduction of HCV RNA from day 7 to 28 from 0.4 log_10_ IU/mL/week in Group C (Standard-of-Care) to 0.6 log_10_ IU/mL/week in Group A or B the study requires at least 35 patients per study arm. The statistical power (chance) for the study to detect a superior effect in Group A or B as compared to C is 80%. Statistical sample-size calculation is based on z-test for differences between proportions and is one-sided with a significance level of 5%.

# Data Quality Assurance

Accurate and reliable data collection will be assured by the investigator. The data collected will be entered into a computer database and subject to quality assurance procedures as dictated by the steering committee. The data collected will be archived for at least 10 years after the termination of the study.

# Publication

Presentations of the study results at conferences and publications in journals will be prepared by the steering committee.

# Study Committees

Chairman: Martin Lagging

Co-Chairman: Johan Westin,

Sweden: Karin Lindahl, Gunnar Norkrans

Denmark: Peer Christensen, Mads Rauning Buhl

Finland: Martti Färkkilä

Norway: Olav Dalgard

**References**

1. Marcellin P, Asselah T, Boyer N. Fibrosis and disease progression in hepatitis C. Hepatology. 2002 Nov;36(5 Suppl 1):S47-56.

2. Seeff LB. Natural history of chronic hepatitis C. Hepatology. 2002 Nov;36(5 Suppl 1):S35-46.

3. Seeff LB, Hoofnagle JH. National Institutes of Health Consensus Development Conference: management of hepatitis C: 2002. Hepatology. 2002 Nov;36(5 Suppl 1):S1-2.

4. EASL International Consensus Conference on Hepatitis C. Paris, 26-28, February 1999, Consensus Statement. European Association for the Study of the Liver. J Hepatol. 1999 May;30(5):956-61.

5. PEGASYS. Summary of Product Characteristics2003 16 July.

6. PEG-IFNalfa-2a. Investigational Drug Brochure2005 July.

7. Sidwell RW, Robins RK, Hillyard IW. Ribavirin: an antiviral agent. Pharmacol Ther. 1979;6(1):123-46.

8. Gilbert BE, Knight V. Biochemistry and clinical applications of ribavirin. Antimicrob Agents Chemother. 1986 Aug;30(2):201-5.

9. Di Bisceglie AM, Conjeevaram HS, Fried MW, Sallie R, Park Y, Yurdaydin C, et al. Ribavirin as therapy for chronic hepatitis C. A randomized, double-blind, placebo-controlled trial. Ann Intern Med. 1995 Dec 15;123(12):897-903.

10. Dusheiko G, Main J, Thomas H, Reichard O, Lee C, Dhillon A, et al. Ribavirin treatment for patients with chronic hepatitis C: results of a placebo-controlled study. J Hepatol. 1996 Nov;25(5):591-8.

11. Fried MW, Shiffman ML, Reddy KR, Smith C, Marinos G, Goncales FL, Jr., et al. Peginterferon alfa-2a plus ribavirin for chronic hepatitis C virus infection. N Engl J Med. 2002 Sep 26;347(13):975-82.

12. Hadziyannis SJ, Sette H, Jr., Morgan TR, Balan V, Diago M, Marcellin P, et al. Peginterferon-alpha2a and ribavirin combination therapy in chronic hepatitis C: a randomized study of treatment duration and ribavirin dose. Ann Intern Med. 2004 Mar 2;140(5):346-55.

13. Pawlotsky JM, Dahari H, Neumann AU, Hezode C, Germanidis G, Lonjon I, et al. Antiviral action of ribavirin in chronic hepatitis C. Gastroenterology. 2004 Mar;126(3):703-14.

14. Loustaud-Ratti V, Alain S, Rousseau A, Hubert IF, Sauvage FL, Marquet P, et al. Ribavirin exposure after the first dose is predictive of sustained virological response in chronic hepatitis C. Hepatology. 2008 May;47(5):1453-61.

15. Cadranel JF, Rufat P, Degos F. Practices of liver biopsy in France: results of a prospective nationwide survey. For the Group of Epidemiology of the French Association for the Study of the Liver (AFEF). Hepatology. 2000 Sep;32(3):477-81.

16. Regev A, Berho M, Jeffers LJ, Milikowski C, Molina EG, Pyrsopoulos NT, et al. Sampling error and intraobserver variation in liver biopsy in patients with chronic HCV infection. Am J Gastroenterol. 2002 Oct;97(10):2614-8.

17. Islam S, Antonsson L, Westin J, Lagging M. Cirrhosis in hepatitis C virus-infected patients can be excluded using an index of standard biochemical serum markers. Scand J Gastroenterol. 2005 Jul;40(7):867-72.

18. Lagging M, Wejstal R, Uhnoo I, Gerden B, Fischler B, Friman S, et al. Treatment of hepatitis C virus infection: updated Swedish Consensus recommendations. Scand J Infect Dis. 2009;41(6-7):389-402.

19. Glue P. The clinical pharmacology of ribavirin. Semin Liver Dis. 1999;19 Suppl 1:17-24.

20. Rao KP, Rahiman MA. Cytogenetic effects of ribavirin on mouse bone marrow. Mutat Res. 1989 Oct;224(2):213-8.

Appendix 1 Child-Pugh Classification of Severity of Liver Disease

|  | **Points Scored for Increasing Abnormality** | | |
| --- | --- | --- | --- |
| **Clinical and Biochemical Measurements** | **1** | **2** | **3** |
| Encephalopathy (grade)^a^ | None | 1 or 2 | 3 or 4 |
| Ascites^b^ | Absent | Slight | Moderate |
| Bilirubin (mg per 100 mL)  SI unit= (µmol/l) | < 2  <34 | 2 – 3  34-51 | >3  >51 |
| Albumin (g per 100 mL) | > 3.5 | 2.8 – 3.5 | < 2.8 |
| INR | < 1.7 | 1.7-2.3 | > 2.3 |

a According to grading of Trey, Burns and Saunders (1996)

b As determined by physical examination alone

1, 2 or 3 points are scored for increasing abnormality of each of the 5 parameters measured.

Grade **A**: Total score of 5 or 6

Grade **B**: Total score of 7 to 9

Grade **C**: Total score of 10 to 15

Appendix 2 PEGASYS Dose Adjustment Guidelines

Specific dose adjustment guidelines for PEGASYS are provided below for post-treatment, neutropenia, thrombocytopenia, elevated serum ALT activities, and depression. It should be kept in mind that other responses by an investigator, in accordance with local guidelines, may be more appropriate, and that an investigator does not have to abide by the dose adjustment guidelines listed below. For other adverse effects considered to be related to PEGASYS, including laboratory abnor­malities, adverse events, and vital sign changes, investigators should utilize the table below labeled “PEGASYS General Dose Reduction Guidelines.” When practicable, abnormal laboratory results should be confirmed as soon as possible following notification of the investigator. If appropriate, downward adjustments in one level decrements (see section 7.3.1) should be considered. The lowest dose of PEGASYS that should be administered is 45 µg. For laboratory and vital signs abnormalities, “severe” is defined as any value requiring intervention, further work-up, or more frequent follow-up.

PEGASYS General Dose Reduction Guidelines

| **Number of Dose Reduction Levels (see Section 7.3.1)** | | | | | |
| --- | --- | --- | --- | --- | --- |
| **Mild** | **Moderate**  **Limited** | **Moderate**  **Persistent** | **Severe**  **Limited** | **Severe**  **Persistent** | **Life-Threatening** |
| 0 | 0 | 0-1 | 0-1 | 1-2 | Stop Drug |

Dose Adjustments for Low Absolute Neutrophil Counts

| **ANC** | **PEGASYS Dose Reduction** |
| --- | --- |
| < 750 cells/mm^3^ | 135 µg |
| < 500 cells/mm^3^ | Withhold treatment until ANC returns to >1000 cells/mm^3^. Resume treatment at 90 µg and monitor ANC |

Dose Adjustments for Low Platelet Counts

| **Platelet Count** | **PEGASYS Dose Reduction** |
| --- | --- |
| < 50.000 cells/mm^3^ | 90 µg |
| < 25.000 cells/mm^3^ | Withhold treatment |

**Appendix 2: PEGASYS Dose Adjustment Guidelines *(Cont.)***

**Dose Adjustments for Elevated Serum ALT**

Fluctuations in abnormalities of liver function tests are common in patients with chronic hepatitis C. As with other alfa interferons, increases in ALT levels above baseline (BL) have been observed in patients treated with PEGASYS, including patients with a virological response.

In patients with progressive ALT increases above baseline, the patient should be closely monitored and if necessary the dose of PEGASYS should be reduced to 135 µg. If ALT increases are progressive despite dose reduction, or accompanied by increased bilirubin or evidence of hepatic decompensation, therapy should be immediately discontinued.

**Dose Adjustments for Depression**

**The responsible physician may consider antidepressants more appropriate than dose reductions.**

| **Depression Severity** | **Initial Management (4-8 weeks)** | | **Depression** | | |
| --- | --- | --- | --- | --- | --- |
|  | **Dose Modification** | **Visit Schedule** | **Remains Stable** | **Improves** | **Worsens** |
| Mild | No change | Evaluate once weekly by visit and/or phone | Continue weekly visit schedule | Resume normal visit schedule | (See moderate or severe depression) |
| Moderate | Decrease PEGASYS dose to 135 µg (in some cases dose reduction to 90 µg may be needed) | Evaluate once weekly (office visit at least every other week) | Consider psychiatric consultation. Continue reduced dosing | If symptoms improve and are stable for 4 weeks, may resume normal visit schedule. Continue reduced dosing or return to normal dose | (See severe depression) |
| Severe | Discontinue PEGASYS permanently | Obtain immediate psychiatric consultation | Psychiatric therapy necessary | | |

**Appendix 3: COPEGUS Intended Dose and Dose Adjustment Guidelines**

| Group A: Intended Ribavirin Dosing | | |
| --- | --- | --- |
| Weight | Ribavirin dose day 0 – day 14* | Ribavirin dose day 15 -  End of Treatment** |
| 45-60 kg | 1600 mg | 800 mg |
| 61-75 kg | 2000 mg | 1000 mg |
| 76-90 kg | 2400 mg | 1200 mg |
| 91-105 kg | 2800 mg | 1400 mg |
| *Dose given TID. **Ribavirin concentration measured on treatment day 28. Dose | | |
| adjustments will be made at the discretion of the treating physician with the goal of | | |
| achieving ≥ 2.5 mg/L (10.25 μmol/L). A new ribavirin concentration is recommended | | |
| after an additional period of 4 weeks if the ribavirin dose is adjusted as above. | | |

| Group B and C: Intended Ribavirin Dosing |  |
| --- | --- |
| Weight | Ribavirin dose day 0 –  End of Treatment ^a,b^ |
| 45-60 kg | 800 mg |
| 61-75 kg | 1000 mg |
| 76-90 kg | 1200 mg |
| 91-105 kg | 1400 mg |
| ^a^No ribavirin concentrations will be measured while on treatment for group C.  ^b^Group B will receive 4 weeks of priming with ribavirin without PEG-IFN. | |

If the ribavirin concentration is below the target 2.5 mg/L (10.25 μmol/L) 28 days after the initiation of ribavirin, the dose of ribavirin should be increased as soon as possible preferably within 2 weeks of measuring the ribavirin concentration if the patient tolerates. The new recommended dose is the previously given dose divided by the concentration of ribavirin measured multiplied by the target concentration, i.e. 2.5 mg/L (10.25 μmol/L) as suggested in the table below. In the event of an increase in ribavirin dosing in Groups A (“Loading”) and B (“Priming”), a new local ribavirin concentration should be evaluated after 4 weeks.

Appendix 4 Intervention Guidelines in the Event of Anemia

The use of erythropoietin (NeoRecormon) ≥3000 IE 3 times week sc initially in the event of anemia is permitted throughout the study at the discretion of the treating physician with the exception if the patient has any of the following exclusion criteria for the use of erythropoietin: platelet count >500,000, risk of thrombosis, or poorly controlled hypertension, especially if hemoglobin decreases persistently to < 10.0 g/dL or in the event of a rapid decrease in hemoglobin exceeding 4.0 g/dL over a 2 week period. If erythropoietin is initiated, a new ribavirin concentration at local lab should be evaluated after 4 weeks in Groups A (“Loading”) and B (“Priming”).

In the event of anemia, dose reductions of ribavirin are permitted in all study arms throughout the study at the discretion of the treating physician.

If hemoglobin decreases to < 8.0 g/dL, ribavirin should be discontinued.

If hemoglobin decreases to < 7.0 g/dL, blood transfusion should be considered at the discretion of the treating physician.

If hemoglobin decreases to < 6.5 g/dL, blood transfusion should be given.

Appendix 5 Instructions for Sample Processing and Shipment for RibaC:

**Please note that all samples should be collected using PPT Vacutainer®Tube before the morning dose of ribavirin because we will be monitoring ribavirin concentrations throughout the study!**

1. **Virology: HCV-RNA (screening visit, day 0 (just prior to the 1^st^ dose of pegylated interferon; day 0 is the day that the first dose of the study medication is given), day 3, day 7 (before the 2^nd^ dose of pegylated interferon), day 28 (before the 5^th^ dose of pegylated interferon), week 12, End of Treatment, Follow-Up week 24)**

**Use PPT Vacutainer®Tube (6ml) BD product:**

- - - - - Draw the tube and immediately mix sample by gentle inversion 10 times; DO NOT SHAKE
        - After mixing, store the PPT tube upright at room temperature until centrifugation
        - Centrifuge the PPT tube at 1100 x g for 10 minutes, swing-out bucket rotor. Blood samples should be centrifuged within two (2) hours of blood collection
        - After centrifugation transfer the tube to the designated local storage laboratory containing a –70°C freezer (if this is not possible use a –20°C freezer)
        - At the storage laboratory transfer the plasma from each PPT tube into three 1 ml tubes.
        - Label the 1 ml tubes
        - Store in –70°C freezer until shipment (if this is not possible store at –20°C)
        - **Please note:** **Samples from screening visit, day 28, week 12, week 24, End of Treatment, and Follow-Up week 24 will also be sent at room temperature to a local laboratory using Roche COBAS TaqMan for HCV-RNA quantification.**

# Shipment

- - - - - Check that labels are in place
        - Place frozen samples in polypropylene container and close it
        - Place polypropylene container into a BioPack
        - Place BioPack into the dry ice in thermal transport box; dry ice should surround the BioPack
        - Send to Department of Virology Göteborg, Sweden upon completion of the study

1. **Parmacokinetics at central lab: Trough concentration of ribavirin (day 1, day 3, day 7, day 28, week 8, week 12, and end-of-treatment) obtained before the morning dose of ribavirin. Please note that for Groups A and B an additional plasma sample will be obtained on day 28 for analysis of ribavirin concentration at a local lab, and if the target concentration is not met, an increase in ribavirin dose in accordance with appendix 3 is recommended, but not mandatory at the discretion of the treating physician. In the event of a change in ribavirin dosing in Groups A (“Loading”) and B (“Priming”), a new local ribavirin concentration should be evaluated after 4 weeks.**

**Use PPT Vacutainer®Tube (6ml) BD product:**

For procedure see section 1. Virology

# Shipment

- - - - - Check that labels are in place
        - Place frozen samples in polypropylene container and close it
        - Place polypropylene container into a BioPack
        - Place BioPack into the dry ice in thermal transport box; dry ice should surround the BioPack

Send to Odense Universitetshospital

Afd Q, bygning 1, 2. Sal Penthouse, blok 6

Sdr. Boulevard 29,

5000 Odense c, Denmark

att: projektsygeplejerske

upon completion of the study

1. **Cholesterol and Triglyceride levels, HbA1c (Fasting sample obtained during the screening period)**

# Use local tubes

- - - - - Send to local lab

1. **Vitamin D (Serum Sample at Screening)**

**Collection of ≥ 2 mL serum (not plasma) and shipment together with ribavirin sample as stated above.**

1. **IL-28B Polymorfism (Screening)**

# Use EDTA tube

- - - - - Freeze and store unseparated in –70°C freezer until shipment (if this is not possible store at –20°C)

# Shipment

- - - - - Place frozen samples in polypropylene container and close it
        - Place polypropylene container into a BioPack
        - Place BioPack into the dry ice in thermal transport box; dry ice should surround the BioPack
        - Send to Department of Virology Göteborg, Sweden upon completion of the study

1. **IP-10 (days 0, 1, 3, 7, and 14, and weeks 8, 18, and 24)**

**Use PPT Vacutainer®Tube (6ml) BD product:**

- - - - - Draw the tube and immediately mix sample by gentle inversion 10 times; DO NOT SHAKE
        - After mixing, store the PPT tube upright at room temperature until centrifugation
        - Centrifuge the PPT tube at 1100 x g for 10 minutes, swing-out bucket rotor. Blood samples should be centrifuged within two (2) hours of blood collection
        - After centrifugation transfer the tube to the designated local storage laboratory containing a –70°C freezer (if this is not possible use a –20°C freezer)
        - At the storage laboratory transfer the plasma from each PPT tube into three 1 ml tubes.
        - Label the 1 ml tubes
        - Store in –70°C freezer until shipment (if this is not possible store at –20°C)
        - **Please note:** **Samples from screening visit, day 28, week 12, week 24, End of Treatment, and Follow-Up week 24 will also be sent at room temperature to a local laboratory using Roche COBAS TaqMan for HCV-RNA quantification.**

# Shipment

- - - - - Check that labels are in place
        - Place frozen samples in polypropylene container and close it
        - Place polypropylene container into a BioPack
        - Place BioPack into the dry ice in thermal transport box; dry ice should surround the BioPack
        - Send to Department of Virology Göteborg, Sweden upon completion of the study

1. **Liver Biopsy: (optional)**

≥4 unstained slides and/or paraffin embedded biopsies

If a liver biopsy is obtained during the screening period, it is encouraged that 1 cm liver tissue be placed in Allprotect Tissue Reagent (Qiagen cat. No. 76405) and immediately frozen at -70°C for later evaluation of mRNA expression of relevant targets reported to be of importance for outcome, e.g. IP-10, IFN-gamma, IFI27, IFIT1, USP18, sialoadhesin, and IFN-lamba.

## Shipment

- - - - - Transport of biopsy slides/paraffin embedded material should be at room temperature. Send to the Department of Virology Göteborg, Sweden after evaluation by local pathologist.
- If liver biopsy in Allprotect Tissue Reagent (Qiagen cat. No. 76405):
  - Place frozen samples in polypropylene container and close it
  - Place polypropylene container into a BioPack
  - Place BioPack into the dry ice in thermal transport box; dry ice should surround the BioPack
  - Send to Department of Virology Göteborg, Sweden upon completion of the study

1. **PBMC’s: Obtained screening (Optional)**

Use Heparin tubes (Green Top, 10 mL):

- Keep at room temperature until separation, which may have a maximum delay of 24 hours between blood collection and separation.
- Spin down the blood for 10 minutes at 2000 rpm.
- Separate blood from plasma and save plasma separately.
- Dilute blood 1:1.5 with sterile PBS.
- Layer 8 mL diluted blood on top (carefully and slowly) of 4 mL Lymphoprep in 15 mL clear conical tubes.
- Centrifuge 30 minutes at 2800 rpm, no brake, at room temperature.
- Collect lymphoprep-plasma interphase and transfer it to a new tube (max 4 mL/tube).
- Add sterile PBS to a total volume of 10 mL.
- Centrifuge 10 minutes at 1600 rpm with brake.
- Discard the supernatant and resuspend the pellet in 10 mL sterile PBS.
- Centrifuge 10 minutes at 1600 rpm with brake.
- Discard the supernatant and resuspend the pellet in 10 mL sterile PBS (pool cells of the same patient at this step).
- Centrifuge 10 minutes at 1600 rpm with brake.
- Discard the supernatant and resuspend the pellet in RPMI-1640 containing 10% fetal bovine serum.
- Count the viable cells using trypan blue.
- Centrifuge 10 minutes at 1600 rpm with brake.
- Discard the supernatant and resuspend the pellet in desired volume (concentration 20 x 10^6^ cells/mL) in RPMI-1640 containing 10% fetal bovine serum.
- Incubate the cells which are left over for at least 15 minutes on ice. In the mean time thaw a tube containing 90% fetal bovine serum and 10% DMSO.
- Add 1:1 the precooled DMSO solution drop by drop and swirl 10 seconds each time so that the final concentration of cells will be 10 x 10^6^ cells/mL.
- Aliquot 1 mL cell suspension per tube.
- Freeze the cells in a cryocontainer at -70°C.
- Transfer the tubes after 24 hours to -135°C or liquid nitrogen.

# Part II: ethics and General Study Administration

# Ethical aspects

## Local Regulations/Declaration of Helsinki

The investigator will ensure that this study is conducted in full conformance with the principles of the “Declaration of Helsinki” (as amended in Tokyo, Venice, Hong Kong, South Africa and Edinburgh) or with the laws and regulations of the country in which the research is conducted, whichever affords the greater protection to the individual. The study must fully adhere to the principles outlined in “Guideline for Good Clinical Practice” ICH Tripartite Guideline (January 1997) or with local law if it affords greater protection to the subject.

## Informed Consent

It is the responsibility of the investigator, or a person designated by the investigator, to obtain written informed consent from each subject participating in this study, after adequate explanation of the aims, methods, anticipated benefits, and potential hazards of the study. For subjects not qualified or incapable of giving legal consent, written consent must be obtained from the legally acceptable representative. In the case where both the subject and his/her legally acceptable representative are unable to read, an impartial witness should be present during the entire informed consent discussion. After the subject and representative have orally consented to participation in the trial, the witness’ signature on the form will attest that the information in the consent form was accurately explained and understood. The investigator or designee must also explain that the subjects are completely free to refuse to enter the study or to withdraw from it at any time, for any reason. The Case Report Forms for this study contain a section for documenting informed subject consent, and this must be completed appropriately. If new safety information results in significant changes in the risk/benefit assessment, the consent form should be reviewed and updated if necessary. All subjects (including those already being treated) should be informed of the new information, given a copy of the revised form and give their consent to continue in the study.

## Independent Ethics Committees/Institutional Review Board

Independent Ethics Committees

This protocol and any accompanying material provided to the subject (such as subject information sheets or descriptions of the study used to obtain informed consent) as well as any advertising or compensation given to the patient, will be submitted by the investigator to an Independent Ethics Committee. Approval from the committee must be obtained before starting the study, and should be documented in a letter to the investigator specifying the date on which the committee met and granted the approval.

Any modifications made to the protocol after receipt of the Independent Ethics Committee approval must also be submitted by the investigator to the Committee in accordance with local procedures and regulatory requirements.

When no local review board exists, the investigator is expected to submit the protocol to a regional committee.

# conditions for modifying the protocol

Protocol modifications to ongoing studies must be made only after consultation between the members of the steering committee.

All protocol modifications must be submitted to the appropriate Independent Ethics Committee or Institutional Review Board for information and approval in accordance with local requirements, and to Regulatory Agencies if required. Approval must be awaited before any changes can be implemented, except for changes necessary to eliminate an immediate hazard to trial subjects, or when the change(s) involves only logistical or administrative aspects of the trial (e.g. change in monitor(s), change of telephone number(s).

# Conditions for terminating the study

The steering committee, the DSMB, and the investigator reserve the right to terminate the study at any time. Should this be necessary, both parties will arrange the procedures on an individual study basis after review and consultation. In terminating the study, the steering committee and the investigator will assure that adequate consideration is given to the protection of the patient’s interests.

# Study documentation, CRFs and record keeping

## Investigator's Files / Retention of Documents

The Investigator must maintain adequate and accurate records to enable the conduct of the study to be fully documented and the study data to be subsequently verified. These documents should be classified into two different separate categories (1) Investigator's Study File, and (2) subject clinical source documents.

The Investigator's Study File will contain the protocol/amendments, Case Report and Query Forms, Independent Ethics Committee/Institutional Review Board and governmental approval with correspondence, sample informed consent, drug records, staff curriculum vitae and authorization forms and other appropriate documents/correspondence etc.

Subject clinical source documents (usually defined by the project in advance to record key efficacy/safety parameters independent of the CRFs) would include patient hospital/clinic records, physician's and nurse's notes, appointment book, original laboratory reports, ECG, EEG, X‑ray, pathology and special assessment reports, signed informed consent forms, consultant letters, and subject screening and enrollment logs. The Investigator must keep these two categories of documents on file for at least 10 years after completion or discontinuation of the study. After that period of time the documents may be destroyed, subject to local regulations.

## Source Documents and Background Data

In case of governmental queries, it is necessary to have access to the complete study records, provided that patient confidentiality is protected.

## Inspections

The investigator should understand that source documents for this trial should be made available to health authority, inspectors after appropriate notification. The verification of the Case Report Form data must be by direct inspection of source documents.

## Case Report Forms

For each patient enrolled, a Case Report Form must be completed and signed by the principal investigator or authorized delegate from the study staff. This also applies to records for those patients who fail to complete the study (even during a pre-randomization screening period if a Case Report Form was initiated). If a patient withdraws from the study, the reason must be noted on the Case Report Form. If a patient is withdrawn from the study because of a treatment-limiting adverse event, thorough efforts should be made to clearly document the outcome.

All forms should be typed or filled out using indelible ink, and must be legible. Errors should be crossed out but not obliterated, the correction inserted, and the change initialed and dated by the investigator or his/her authorized delegate. The investigator should ensure the accuracy, completeness, legibility, and timeliness of the data reported to the sponsor in the CRFs and in all required reports.

# monitoring the study

Monitoring of the study will be performed by a qualified monitor provided by Roche AB Sweden, on delegation basis.

The monitor will contact and visit the investigator regularly and will be allowed, on request, to inspect the various records of the trial (Case Report Forms and other pertinent data) provided that patient confidentiality is maintained in accord with local requirements.

It will be the monitor's responsibility to inspect the Case Report Forms throughout the study, to verify the adherence to the protocol and the completeness, consistency and accuracy of the data being entered on them. The monitor should have access to laboratory test reports and other patient records needed to verify the entries on the Case Report Form. The investigator (or his/her deputy) agrees to cooperate with the monitor to ensure that any problems detected in the course of these monitoring visits are resolved.

# confidentiality of trial documents and subjecT records

The investigator must assure that subjects’ anonymity will be maintained and that their identities are protected from unauthorized parties. On CRFs or other documents submitted to the data managers, subjects should not be identified by their names, but by an identification code. The investigator should keep a subject enrollment log showing codes, names and addresses. The investigator should maintain documents e.g., subjects’ written consent forms, in strict confidence.

# publication of data and Protection of trade secrets

The results of this study may be published or presented at scientific meetings. Authorship will be determined by the steering committee.
